# Immune System Effects of Insulin-Like Peptide 5 in a Mouse Model

**DOI:** 10.3389/fendo.2020.610672

**Published:** 2021-01-14

**Authors:** Brett Vahkal, Sergey Yegorov, Chukwunonso Onyilagha, Jacqueline Donner, Dean Reddick, Anuraag Shrivastav, Jude Uzonna, Sara V. Good

**Affiliations:** ^1^ Department of Biology, University of Winnipeg, Winnipeg, MB, Canada; ^2^ Department of Immunology, University of Manitoba, Winnipeg, MB, Canada

**Keywords:** insulin-like peptide 5, relaxin family peptide receptor 4, immune markers, immune system, metabolic syndrome, gut peptide hormone

## Abstract

**Introduction:**

Insulin-like peptide 5 (INSL5) is a peptide hormone with proposed actions in glucose homeostasis and appetite regulation *via* its cognate receptor, relaxin family peptide receptor 4 (RXFP4). Here, we look for evidence for their involvement in the immune system using a mouse model.

**Methods:**

*In silico analyses:* we queried public databases for evidence of expression of INSL5-RXFP4 in immune system tissues/cells (NCBI’s SRA and GeoProfiles) and disorders (EMBO-EBI) and performed phylogenetic footprinting to look for evidence that they are regulated by immune-associated transcription factors (TFs). *Experimental analyses:* We characterized the expression and correlation of INSL5/RXFP4 and other immune system markers in central and peripheral immune organs from C57/bl6 mice in seven cohorts. We tested whether fluctuations in circulating INSL5 induce an immune response, by injecting mice with 30 μg/kg of INSL5 peptide in the peritoneum, and examining levels of immune markers and metabolic peptides in plasma. Lastly, we quantified the expression of *Rxfp4* in T-cells, dendritic cells and cell lines derived from human and mouse and tested the hypothesis that co-incubation of ANA-1 cells in INSL5 and LPS alters cytokine expression.

**Results:**

We find *Insl5* expression only in thymus (in addition to colon) where its expression was highly correlated with *Il-7*, a marker of thymocyte development. This result is consistent with our *in silico* findings that *Insl5* is highly expressed in thymic DP, DN thymocytes and cortical TEC’s, and with evidence that it is regulated by thymocyte-associated TF’s. We find *Rxfp4* expression in all immune organs, and moderately high levels in DCs, particularly splenic DCs, and evidence that it is regulated by immune-associated TF’s, such as STAT’s and GATA. *Systemic effects*: We observed significantly elevated concentrations of blood GLP-1, GIP, GCG and PYY following intraperitoneal injection of INSL5, and significantly altered expression of cytokines IL-5, IL-7, M-CSF, IL-15, IL-27 and MIP-2. *Immune cell effects*: Incubation of ANA-1 cells with INSL5 impeded cell growth and led to a transient elevation of IL-15 and sustained reduction in IL-1β, IL-6 and TNFα.

**Conclusion:**

We propose that INSL5-RXFP4 play a novel role in both central and peripheral immune cell signaling.

## Introduction

Insulin like peptide 5 (INSL5) is a novel peptide hormone belonging to the relaxin (RLN)/INS/IGF superfamily that plays roles in neuroendocrine processes *via* its cognate receptor, relaxin family peptide receptor 4 (RXFP4) (1–3). Primary tissues of expression for *Insl5* are the L-cells of the distal colon, hypothalamus, kidney, thymus and reproductive tissues; while *Rxfp4* is expressed in the colon and subtending vagus nerve efferents, as well as in the cerebellum, reproductive tissues and kidney: (4, 5) GTEX consortium). Collectively these tissues of expression correlate well with current hypotheses that INSL5 a) is a gut hormone that modulates glucose metabolism either directly or indirectly *via* gluconeogenesis (6–8); b) is an orexigenic hormone that influences satiety, through both gut-nervous system and hypothalamus-cerebellum cross-talk with RXFP4 (9); c) plays roles in female and male fertility (6, 10, 11), and d) together with RXFP4, are markers of colorectal and breast cancer progression (12–15).

Since the discovery of INSL5 in 1999, there have been a few indications that it may also play a role in the immune system. For example, *Insl5* and/or *Rxfp4* transcripts have been found in immune system tissues such as thymus, leukocytes, bone marrow and lymph nodes (5), and a study in lean and obese men and women, found a negative correlation of INSL5 with the pro-inflammatory biomarker C-reactive protein (CRP) (16). Here, we explore, for the first time, the hypothesis that INSL5 and RXFP4 play roles in the immune system using both *in silico* and experimental methods. To this end, we mined public databases for evidence of the expression of *Insl5/Rxfp4* in immune tissues/cells and performed phylogenetic footprinting to look for evidence that they are regulated by immune-associated transcription factors (TFs). Next, we used experimental methods to test the hypothesis that *Insl5/Rxfp4* are expressed in both central and peripheral immune tissues. To this end, we quantified expression of *Insl5* and *Rxfp4* using qPCR in central and peripheral immune tissues (thymus, blood, bone marrow, colon and spleen) in C57/Bl6 mice stratified by age (from 3 weeks to >12 months), and tested the hypothesis that they exhibit age-dependent expression in thymus by examining both their change in expression over age cohorts and the correlation of their expression with other genes known to be involved in thymus development or involution.

Finding evidence of broad expression of *Rxfp4* in immune tissues and subsets, we moved to an *in vivo* model and tested whether intraperitoneal (i.p.) injection of INSL5 in C57/Bl6 mice resulted in changes in the levels of circulating immune system mediators, and/or metabolic peptides, given the known insulinotropic effects of INSL5. Finally, we looked at the cellular level, and hypothesized that INSL5 may convey signals through RXFP4 expression on major immune cell subsets. Accordingly, we assessed the expression of *Rxfp4* in CD4+ T-cells and bone-marrow derived and splenic DCs (BMDC, sDCs), as well as in five cell lines derived from human and mouse tissues and then performed an experiment in which we pre-incubated mouse ANA-1 bone-marrow derived macrophages (BMDM) in INSL5 for up to 18 h, and then stimulated cells with LPS and screened for expression of focal cytokines.

Our results indicate a broader expression of *Insl5/Rxfp4* in immune system tissues and cells than previously described. We find evidence that *Insl5* plays a role in the development of thymocytes based on *in silico* datamining of RNA-seq datasets and phylogenetic footprinting analyses and from experimental data that show a correlation of *Insl5* and *Il-7* in age-stratified cohorts of C57/Bl6 mice. In the peripheral immune system, we show that *Rxfp4* is expressed in diverse immune cell subsets of the innate immune system, particularly splenic dendritic cells, and find that INSL5 treatment alters levels of pro-and anti-inflammatory cytokines both *in vitro* and *in vivo;* most notably a decrease in pro-inflammatory cytokines IL-6 and IL-1β. Thus, we conclude that INSL5-RXFP4 play a novel role in the immune system.

## Methods

### *In Silico* Analyses

*NCBI-SRA:* We used the human and mouse full length coding sequences (Human *Insl5* ENST00000304526, Human *Rxfp4* ENST00000368318; Mouse *Insl5*, ENSMUST00000106869 Mouse *Rxfp4* ENST00000368318), to query immune-relevant RNA seq datasets in the sequence read archive (SRA) for relevant tissues and cellular subsets (www.ncbi.nlm.nih/SRA). Only experiments for which fragments from the full gene length were identified in at least 5 experiments were counted as positive hits. *Phylogenetic footprinting:* We employed phylogenetic footprinting to identify potential transcription factor binding sites (TFBS’s) in the regulation of *Insl5* and *Rxfp4* by scanning promoter/intronic/UTR regions of each gene with positional weight matrices (PWMs) of known TF’s (17, 18). Putative regulatory regions were identified by aligning 1,000 bp upstream, the intron and 3’ UTR for *Insl5* or 3,000 bp upstream for *Rxfp4* for both human and mouse using mVISTA’s Shuffle-LAGAN glocal alignment algorithm (19, 20) and viewed using a 100 bp sliding window and a 70% conservation threshold on a percent identity plot (PIP) (17, 21). Conserved DNA sequences were extracted and submitted to PROMO [http://alggen.lsi.upc.es/cgi-bin/promo_v3/promo/promoinit.cgi?dirDB=TF_8.3], to identify potential TFBS’s based on the TRANSFAC database (22–24), and candidate TF’s were grouped into nine biological categories (25) ding - those with immune-related functions were selected for further analyses. *Search for pan-vertebrate conservation of immune-related TFBS’s and experimental support of TF’s from the ENCODE project.* The union of all immune-associated TF’s predicted to be in regulatory regions for *Insl5* or *Rxfp4* were used as queries for the pan-vertebrate TFBS finder tool Contra V3 using a core and similarity stringency of 0.95 and 0.85 (26). The data were then visualized in the UCSC genome browser (http://genome.ucsc.edu) with the phastConsElements100 vertebrate alignment table and the ENCODE regulation tracks for H3K4Me1 marks (open chromatin), DNase I hypersensitivity clusters and confirmed Chip-Seq TFBS’s of one of the 181 TF’s in one or more cell lines interrogated by the ENCODE consortium (27).

### Experimental Work

*Animals:* The effect of aging on *Insl5/Rxfp4* gene and protein expression was determined by qPCR since no validated antibody is available for purchase. For this purpose, 22 C57BL/6 strain (C57BL/6NCrl, Charles River Laboratories, Wilmington, MA) mice were used, 19 belonged to cohorts between three and 16 weeks old, and three were retired breeders aged between 52–78 weeks. Mice were held at the University of Winnipeg animal care facility under standard protocol of housing, feeding and euthanasia. Following euthanasia, mice were dissected and samples of thymus, blood (drawn from the left ventricle), bone marrow (flushed out from tibia with 1X sterile PBS, and then centrifuged at 3,000 g for 10 min), liver, spleen, colon, and testes were bi-sectioned and flash frozen in liquid nitrogen. For the cell line experiments on mouse bone marrow derived (BMDCs) and splenic dendritic cells (sDCs), 8–10-week old C57BL6 male mice were used and housed in University of Manitoba animal care facility. All experiments were carried out in accordance with UACC guidelines and regulations, as described in the animal care protocols for the mice at University of Winnipeg (protocol no. 00284/04606) and the University of Manitoba (protocol no. 14-014) following approval by the Animal Care committees at both institutions.

#### *Insl5/Rxfp4* Expression in Mouse Tissues

The expression of *Insl5* and *Rxfp4* across age cohort and in different tissues was assessed using qPCR. Total RNA was extracted from colon, thymus, and spleen using the RNeasy Plus Universal Mini Kit (QIAgen) following the manufacturer’s protocol, from blood and bone marrow using a phenol-chloroform protocol described in (28), and from cell culture using Trizol as per the manufacturers protocol (ThermoFisher). cDNA was synthesized using 2 μg of total RNA for tissues or 1 μg of RNA for cell culture samples, using Superscript III (ThermoFisher) and random primers (Bio-Rad) in a final reaction volume of 20 ul, and diluted to 10 ng RNA/μl and stored at -20°C until use. Quantitative PCR was performed using FAM-labelled probes (Bio-Rad) for our focal genes *Insl5, Rxfp4*, and for *Ghr* and *Foxn1* (Ensembl IDs: ENSMUSG00000066090, ENSMUSG00000049741, ENSMUSG00000055737, ENSMUSG00000002057 respectively), which are associated with maintenance of thymic architecture (29–31). Additionally, we screened for expression of cytokine *lL-7*, which is involved in the development/selection of thymocytes between double-negative (DN) and double positive (DP) stages in the thymic cortex and differentiation of T-cells in the thymic medula using primers sequences obtained from Ortman et al. (32). Two reference genes were employed, *Ubc-2* and *18S rRNA* using published primers, which have been shown to exhibit stable expression across immune system tissues and cell types (33, 34) (listed in **Table S1**). The efficiency and specificity of the qPCR primers and probes were validated using gel electrophoresis, qPCR melt curve visualization and DNA sequencing of PCR products [see (28)]. For qPCR, 10 μl reaction volumes were prepared using SsoAdvanced Universal SYBR Green Master Mix (Bio-Rad) for primers, and SsoAdvanced Universal Probes Supermix (Bio-Rad) for FAM-labelled probes; reactions were run in a CFX Connect thermocycler (Bio-Rad) using an annealing/extension temperature of 60°C and a standard cycling protocol. Expression of genes across time points and tissues was analysed using the comparative threshold cycle (Ct), ΔΔCt method (35) by first calibrating the expression of genes relative to the geometric mean of the Ct value of the two reference genes in each tissue (i.e. ΔCt_tissue1GOI_ = Ct_(tissue1)_(geometric mean reference genes) – Ct_(tissue1)_(target gene)) and then relative to a calibrator tissue (for single-gene analyses of Rxfp4 across immune tissues) or time-point for the time-based analyses (3 weeks). Additionally, correlations between transcript abundance of *Insl5* and *Rxfp4* with each other and *Il-7, Ghr* and *Foxn1* were performed by converting the raw qPCR values to molecule counts using the R-package MCMC.qPCR (36).

#### Systemic Effects of INSL5

Given the presence of *Insl5* and, more so, *Rxfp4* in central and peripheral immune tissues, we next sought to assess whether mice exhibited a peripheral immune response to intraperitoneal injection of *Insl5* peptide in two pilot experiments (n = 8 and n = 14, respectively). Three days prior to the start of the experiment, mice were subjected to 70% calorie restriction (9); at which point *ad libitum* chow access was replaced with 3.4 g of chow delivered each morning throughout the experiment for a maximum of five days. On the fourth day, experimental mice were i.p. injected with INSL5 peptide (Phoenix Pharmaceuticals, 035-40A) at a concentration of 30 μg/kg of mouse weight, diluted in sterile 1x PBS (BioShop) for a total volume per injection of 100 μl (8, 9). Control mice were injected i.p. with 100 μl of 1 x PBS as time-matched vehicle control. Following terminal anesthesia, 200 μl was sampled from left ventricle of the heart at 6, 24, and 48h post injection and suspended in 5% v/v 0.5 M EDTA (disodium dehydrate, ph 7.4, Amersham) for anti-coagulation and kept on ice until centrifugation. Blood was spun for 10 min at 1,000 g to separate plasma. Serum was prepared according to Eve Technologies (Calgary, Canada, see **Table S4**). A discovery cytokine panel of 32 cytokines and chemokines of broad relevance, and a TH-17 panel including 11 cytokines were analyzed (n = 14, **Supplementary File 3**). For the metabolic assay, 11 peptides were analysed (n = 8, **Supplementary File 3**) including amylin (active), C-Peptide 2, GIP (total), GLP-1 (active), Ghrelin (active), Glucagon, Insulin, Leptin, PP, PYY, and Resistin. The effect of INSL5 injection on plasma cytokine and metabolic markers, was assessed using general linear models (GLM); variables were box-cox transformed, and treatment included as a fixed effect using the control group as reference (1,0 coding). Type III sums of squares were used to test for global effects of the treatment using F-statistics and post-hoc Tukey tests used to identify which treatment group (level) exhibited significantly different expression from the control. *Cytokine levels in splenic T-cells:* Single cell suspension of spleens from i.p. injected mice were stained *ex vivo* for expression of CD3, CD4, CD8, IL-17, IL-10, TNFα, and IFN-γ. Fluorochrome-conjugated anti-mouse antibodies (mAbs) were obtained from eBioscience (San Diego, CA). For intracellular cytokine staining, after surface staining, cells were fixed in 2% paraformaldehyde (Sigma-Aldrich), permeabilized with 0.1% saponin (Sigma-Aldrich) in staining buffer, and stained with specific fluorochrome-conjugated mAbs against IL-17, IL-10, TNFα, and IFN-γ. Cells were acquired using BD FACS Canto II (BD Bioscience, Mississauga, ON, Canada) and analyzed using FlowJo software (Tree Star, Ashland, OR). Additional graphs and statistical analyses were done using GraphPad Prism software version 8.0 (GraphPad, La Jolla, CA).

#### INSL5 Expression in and Effect on Immune System Cell Subsets

We next examined whether *Rxfp4* is expressed in immune cell subsets, including bone-marrow derived and splenic DCs, as well as five macrophage and cancer cell lines from mouse or human, respectively. *BMDCs:* Bone marrow cell culture was established for production of precursor DCs (immature) by plating bone marrow single cell suspension on five individual petri dishes at 2 million cells per plate for four time points at 1, 3, and 7 days post-differentiation with complete RPMI (DMEM supplemented with 10% heat-inactivated fetal bovine serum, 2 mmol L-glutamine, 100 U/ml Penicillin, and 100 μg/ml streptomycin) and GM-CFS (20 ng/ml; PeproTech, Rocky Hill, NJ, USA). Additional complete RPMI and GM-CFS (20 ng/ml) was added on day 3. Additional cells from day 7 were counted and divided into three treatments of 1 million cells with five replicates: unstimulated immature DC’s, or stimulated with LPS (100 ng) for 6 or 24 h. RNA was collected from all samples and screened for the presence of *Rxfp4* and *cd14*, a marker of DC differentiation, for all cell fractions. *Dendritic cells:* DCs were isolated from three spleens following the protocol outlined by EasySep™ Mouse CD11b Positive Selection Kit II (STEMCELL) and the purity of the DC fraction assessed by flow cytometry by gating on CD11c. *Cell lines.* We screened five cell lines for expression of *Insl5* and *Rxfp4* including two macrophage cell lines from mice (ANA-1 and BALB/c), and three cancer cell lines from human (Jurkat, SupT, MCF-7). Cells were grown to a confluence of 2.0x10^6^ cell, RNA extracted using Trizol (ThermoFisher) using the manufacturer’s direction, and qPCR performed with pre-designed SYBR primers (purchased from Sigma-Aldrich) for the focal genes (*Insl5* and *Rxfp4*) and two house-keeping genes per species (*ubc*-2 and *S18* in mouse and *GAPDH* and *RPLPO* in human) using SsoAdvanced Universal SYBR Green Master Mix (Bio-Rad) as above. The relative expression of *Insl5* and *Rxfp4* was assessed relative to the house-keeping genes as: ΔCt_tissue1GOI_ = Ct_(tissue1)_ (geometric mean reference genes) – Ct_(tissue1)_(target gene).

##### Effect of Insl5 Incubation on Cytokine Expression in ANA-1 Mouse Macrophages

Next, we examined the effect of INSL5 on growth rate of the ANA-1 cells and their expression of cytokines by treating cells with 100 nM INSL5 and 1 ug/ml of LPS simultaneously (co-treatment), or pre-treating cells with INSL5 for 3, 6, 12, or 18 h prior to stimulation with LPS, and included a treatment with LPS alone as control. Each of the six treatments was established in triplicate, and the experiment run for two durations: stimulating cells in INSL5/LPS or LPS alone for 12 or 36 h. At the end of the experiment, the number of viable cells at 12 or 24 h was re-counted on a cell counter, RNA extracted and qPCR performed as above to assess changes in the expression of six cytokines: IL-1β, IL6, IL10, TNFα, and Il-5, IL-15, the first six being important immune modulators and the latter two exhibited significant changes in the injection experiment. The relative expression of cytokines was assessed using a Bayesian analyses of qRT-PCR data as implemented in the R package mcmc.qpcr (36). This approach is robust to handling genes with low expression (typical of many cytokines). It employs a generalized linear mixed model based on a prior Poisson-lognormal distribution of values, and then calculates a Bayesian z-score (the mean of the posterior divided by its standard deviation) to perform a standard z-test and derive two-tailed p-values. Differences in viable cell counts among treatments were calculated using the glm function in R and the log2 fold-difference of cytokines between each treatment group and the control was assessed by correcting the above Bayesian two-tailed p-values for difference among treatments with a Bonferonni global correction for multiple testing (α = 0.05/75 test, p < 0.00067) from the summary$genewise output in MCMC.qpcr (36). All analyses were conducted in R v4.0.2.

## Results

### *Insl5* and *Rxfp4* Signatures Are Present in Immune System Organ-Derived RNA-Seq Data

*Insl5* expression was restricted to the thymus and specific lymphocyte subsets (**Table 1** and **Table S2**). In the thymus, *Insl5* was highly expressed in thymocytes overall, especially in DP and DN thymocytes but also in thymic epithelial cells (TECs) – cortical TEC’s (cTECs) and to a lesser extent in medullary TEC’s (mTECs), and thymic natural Th17 cells (**Table 1**). Peripherally, *Insl5* was expressed at low levels in naïve and Th1 CD4+ T-cells, CD8+ T-cells and intestinal intraepithelial lymphocytes (IELs). Compared to *Insl5*, *Rxfp4* was more broadly expressed across all queried immune tissues and cell types (**Table 1**, **Table S2**). Substantial (> 10 reads per sample) *Rxfp4* expression was detected in blood, spleen, lymph node, and bone marrow, while lower levels were also found in thymus. Of the queried cell types, hematopoietic stem cells, splenic CD8+ memory T-cells and monocytes had the highest levels of *Rxfp4* expression, while lower *Rxfp4* expression was detected across most other cell types.

**Table 1:** *Insl5* and *Rxfp4* expression in human (H) and mouse (M) immune system tissues and cells.

	INSL5		RXFP4	
Organism ⇒	H	M	H	M
**IMMUNE SYSTEM ORGANS**				
**Bone marrow**	–	–	+	+
**Lymph node **	–	–	–	+
**Spleen**	–	–	+	+
**Thymus (8-10 week old mice)**	na	++	na	^
**Blood**	–		+	+
**Colon**	++	++	+	+
**IMMUNE CELL PRECURSORS**				
**Hematopoietic stem cells**	–	–	++	++
**Thymocytes (DP, DN)** **(5-6 week old mice)**	na	++	na	^
**Thymic epithelial cells** **cTECs/mTECs**	na	+/^	na	+/^
**B-cells (naïve)**	–	–	–	–
**MATURE IMMUNE CELLS**				
**Intestinal intraepithelial lymphocytes**	na	^	na	–
**CD8+ T cells (memory)**	–	^	++	+
**B-cells (activated)**		–	+	+
**Monocyte (classical, CD40+)**	–	–	++	+
**Dendritic cells**	–	–	+	+
**NK cell (CD16+)**	–	–	+	+
***CD4+ T cells***				
**Naïve**	^	+	^	+
**Th17**	–	–	+	^
**Thymic Th17**	na	+	na	^
**TFH**	–		+	
**Treg**	–	–	+	^
**Th1**	na	^	na	–
**Th2**	na	–	na	–

“-” not found, ^ - a few reads identified, not necessarily representing the full gene “+” <5 full length reads identified per experiment, “++” >10 full length reads identified per experiment, na, data not available.

Data retrieved from the RNA sequence experiments available through the NCBI Sequence Read Archive (SRA) database (see **Supplementary File 1** for list of SRA project ID’s associated with the database mining).

### Phylogenetic Foot-Printing of *Insl5* and *Rxfp4* Regulatory Regions Identifies Immune-Related Transcription Factors

**Figure 1:**
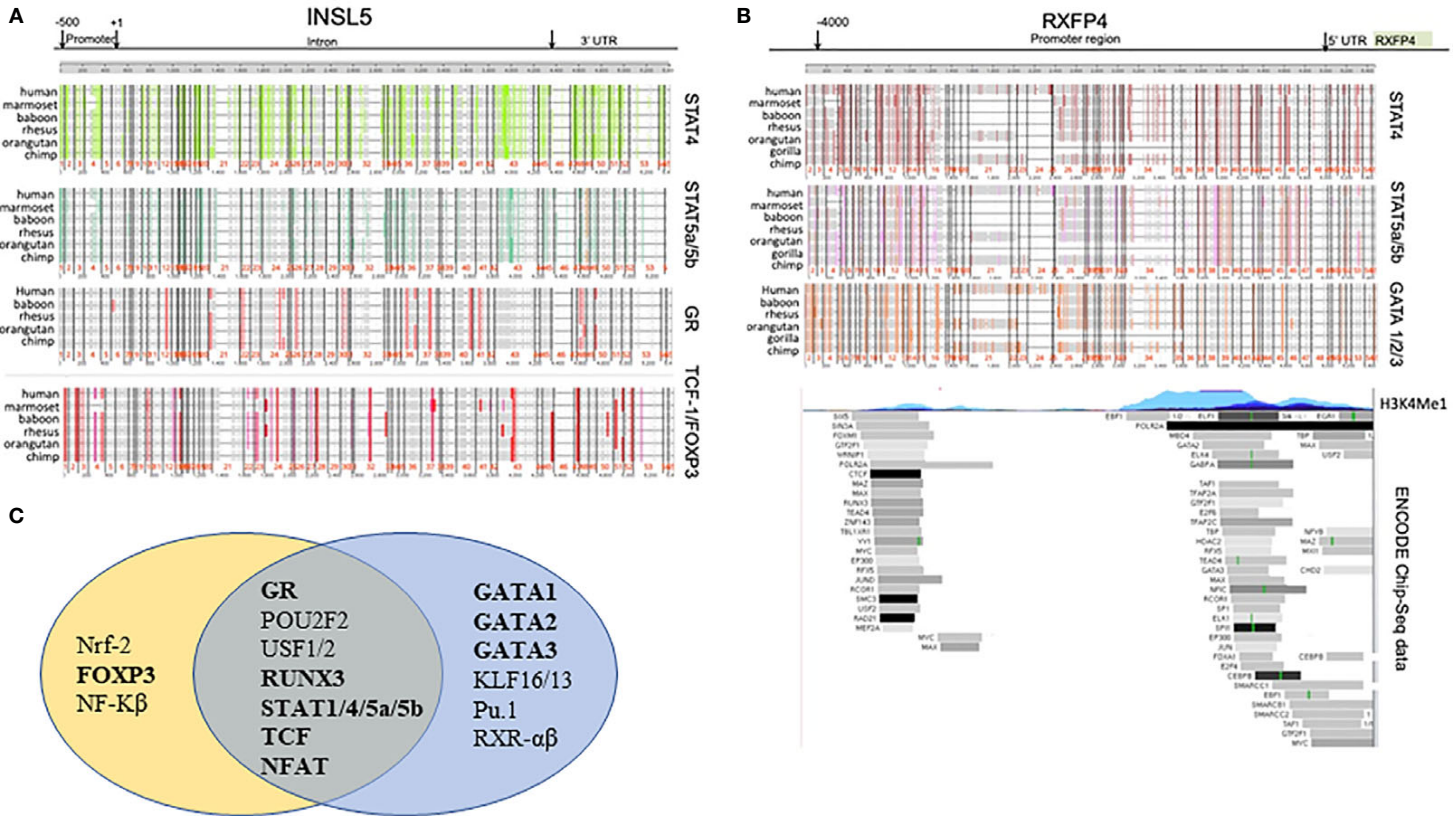
Phylogenetic footprinting analysis of the regulatory regions neighbouring *Insl5* and *Rxfp4*, as well as experimental support for regulatory regions and TF’s associated with the *Rxfp4* gene (ENCODE project). **(A)** Evidence for conserved TFBS motifs in regulatory regions of Insl5 across vertebrates relative to the gene in humans. Upper panels – conserved motifs for STAT4, STAT5a/STAT5b, GR and TCF-1/FOXP3 in the core promoter (500bp) intron, and 3’UTR of Insl5 (full details and other TF’s presented in supplementary data). **(B)** Evidence for conserved TFBS motifs in regulatory regions of *Rxfp4*. Upper panel, Conserved TFBS in 4000 bp region upstream and in the 5’UTR relative to the gene in humans for STAT4, STAT5a/STAT5b and GATA 1/2/3. Middle panel H3K4Me1 sites showing evidence of open chromatin. Lower panel – Experimental evidence for binding of TF’s examined by ENCODE (181 in total) in multiple cell lines and tissues (data last accessed January 2020). **(C)** Venn Diagram showing potential transcription factors involved in Immune/Haematopoietic processes for *Insl5* and *Rxfp4*.

**Figure 2:**
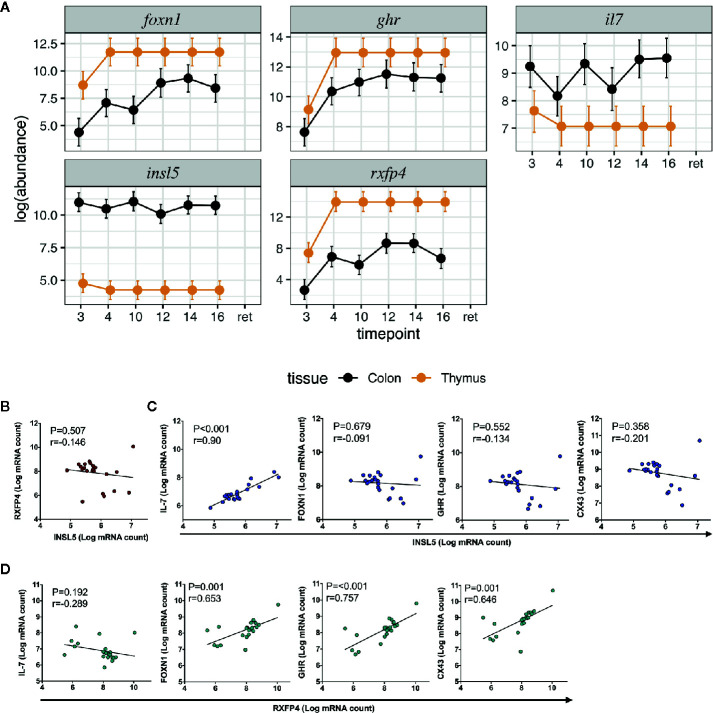
Expression and correlation profiles of *Insl5, Rxfp4, FoxN1, Ghr*, and *Il-7* in mouse immune tissues; **(A)** Temporal and spatial expression of *Insl5*, *Rxfp4, Ghr, and Il-7* in colon and thymus of mice aged 3–16 weeks and retired breeders, **(B)** Correlation of *Insl5* and *Rxfp4* expression in thymus, **(C)** Correlation *of Insl5 vs Il-7, FoxN1*, and *Ghr* in thymus, **(D)** Correlation of *Rxfp4* vs *Il-7*, *FoxN1*, and *Ghr* in thymus. Correlation analyses were done using R-package MCMC.qPCR by converting the raw qPCR values to molecule counts.

Based on the *in silico* analyses of CNE’s between human and mouse, three conserved regions for both *Insl5* and *Rxfp4* were identified (**Supplementary File 2**, **Figures 2A, B**); the subtending sequences were extracted and submitted to promo for TF identification. Fifty-one TFs were identified as potentially regulating the expression of *Rxfp4* and/or *Insl5* (**Supplementary File 2**); the majority of them belonged to the zinc-finger, helix-turn-helix and winged helix-turn-helix DNA-binding families (**Supplementary File 2**). Genomic rearrangement of the regions flanking *Insl5* in the murid lineage rendered it difficult to identify CNE’s for *Insl5*, and most identified CNE’s for *Insl5* were intronic (**Supplementary File 2**, **Figure 2A**). Twenty TF’s were identified as having potential immune/haematopoietic functions and submitted to ContraV3 for further pan-vertebrate analyses using the gene regions in human as reference (**Supplementary File 2**). For *Insl5*, there was strong *ab initio* support that the glucocorticoid receptor (GR), STAT4, STAT5a/STAT5b, T cell Factor 1 (TCF-1), c-ETS-1, FOXP3, and STAT1 (**Figure 1A**, **Supplementary File 2**), are potential TF’s, and some support for binding to RUNX3, NF-AT. The genomic region housing *Insl5 i*s not characterized for H3K4Me1 marks (open chromatin), DNase I hypersensitivity clusters, or Chip-Seq experiments by the ENCODE project. For *Rxfp4*, there was strong support for TFBS motifs that could bind: GR, STAT1, STAT4, STAT5a/5b, TCF-1, c-ETS1, GATA1-3 (**Figure 1B**, **Supplementary File 2** for full details of all sites and support). Of these TF’s, GR, TCF-1, STAT4 and STAT5b are not included in the ENCODE 181 TF Chip-Seq data, but there is Chip-Seq evidence that GATA2 and GATA3 are TF’s for *Rxfp4* in HUVEC (GATA2) and T-47D (GATA3) cell lines—the former an umbilical cord epithelial cell line and the latter an epithelial cancer cell line. Thus, it appears that GR, STAT4, STAT5a/5b and TCF-1 may regulate both *Insl5* and *Rxfp4*, while FOX-P3 may additionally regulate *Insl5* and GATA1, 2, and 3 *Rxfp4* (**Figure 1C**), the latter of which is supported by experimental evidence from the ENCODE project.

### *Insl5* and *Rxfp4* are Variably Expressed in Mouse Immune Tissues

**Figure 3:**
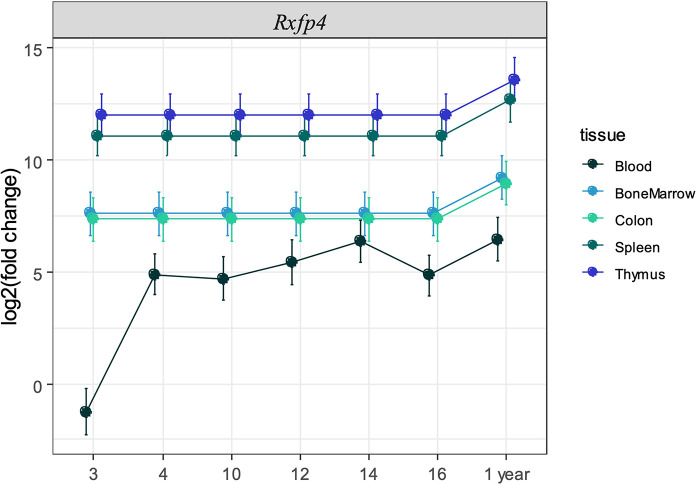
Expression of *Rxfp4* across immune-related tissues and cells. Log2 fold change in expression of *Rxfp4* relative to the housekeeping genes *Ubc-2, 18S rRNA*, and relative to the expression in blood in three-week old mice for each tissue.

We next sought to validate the presence of *Insl5* and *Rxfp4* in blood, thymus, bone marrow, colon, and spleen in C57/Bl6 mice stratified by age (from 3 weeks to >12 months). Consistent with the RNA-seq profiles, *Insl5* mRNA was detected only in thymus and colon (**Figure 2A**, **Figure S2**). However, total thymic expression of *Insl5* was relatively uniform and lower than that of thymic markers *Il-7* and *FoxN1*, and lower than *Rxfp4* (**Figure 2A**). In thymus, the expression of *Rxfp4* was similar to that of thymic markers *FoxN1* and *Ghr*, lowest at 3 weeks, but then increasing markedly and remaining at similar levels in older animals (**Figure 2A**). *Rxfp4* mRNA was detected in all immune system tissues, exhibiting the highest expression in thymus and spleen, followed by bone marrow and then blood (**Figure 3**, **Figure S1**). There was no significant change in expression within a tissue over time, except in blood, for which expression of *Rxfp4* was significantly lower in 3-week old mice relative to all other age cohorts (**Figure 3**). We examined the correlation in expression of *Insl5* and *Rxfp4* with each other and three markers of thymocyte development. While no correlation was observed between *Insl5* and *Rxfp4* (**Figure 2B**), *Insl5* expression was highly correlated with that of *Il-7* (r = 0.9, p < 0.001) (**Figure 2C**), and expression of *Rxfp4* correlated with that of *FoxN1* (r-0.65, p < 0.001) and *Ghr* (r = 0.75, p < 0.001) (**Figure 2D**).

### INSL5 Treatment Changes Levels of Immune and Metabolic Markers in Mouse

**Figure 4:**
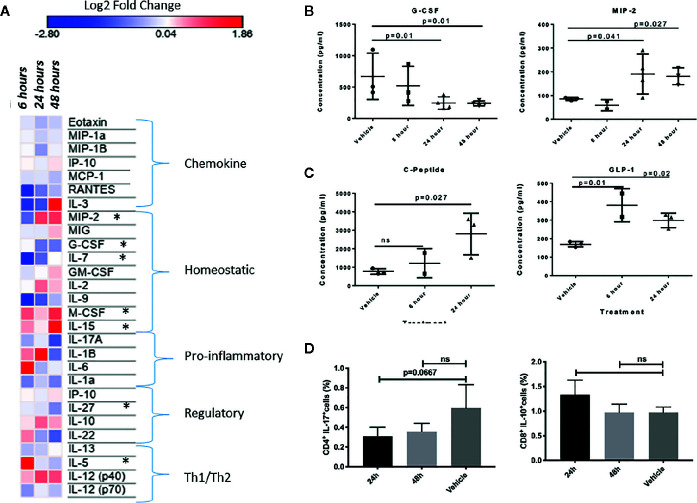
**(A)** Heat-map visualization of changes to 27 chemokines and cytokines following *in vivo* injection of INSL5. Contrast analyses comparing control (PBS injection) to three post-injection intervals (6, 24, and 48 h) found significant differences in seven cytokines over time (MIP-2, G-CSF, IL-7, M-CSF, IL-15, IL-27, IL-5). **(B)** Difference in plasma concentration of G-CSF and MIP-2 in control (PBS injected) from treated (INSL5 injected) mice 6, 24, and 48 h intraperitoneal injection. **(C)** Mice injected with INSL5 showed significantly different levels of GIP, and insulinotropic hormone GLP-1. **(D)** Decreased IL-17 and increased IL-10 levels 24 h post-INSL5 i.p. injection in mouse splenic CD4+ and CD8+ T-cells, respectively. Percent positive cell populations detected using BD FACSCanto-II. Statistical analyses using one-way ANOVA, multiple comparisons.

We reasoned that if INSL5-RXFP4 axis is indeed implicated in immune system signaling as suggested by *in silico* and expression analyses, then INSL5 fluctuation may have detectable effects on systemic markers of the immune response. To test this, we performed INSL5 injections and assessed the changes in levels of circulating immune mediators and metabolic peptides. In support of the insulinotropic features of INSL5, we observed significantly elevated concentrations of blood GLP-1 (6 h, 24 h, p.i.) and C-peptide (24 h, p.i.) (**Figure 4C**) relative to vehicle, as well as Insulin (24 h, p.i.) (**Table S6**). Significant elevations were seen also for glucagon (24 h, p.i., **Table S6**), which inhibits insulin secretion, as well as for PP (6 h p.i), PYY (24 h p.i.) and Amylin (24 h, p.i.) (**Table S6**). Of the cytokines surveyed, 27 produced sufficient levels for analyses: of these, significant alterations were seen for MIP-2 and G-CSF (MIP-2 increased and G-CSF decreased at 24 and 48 h p.i.), while treatment level effects were seen for IL-7 and IL-5 (both decreased at 6 h p.i.), eotaxin, (decreased at 24 h p.i.) and M-CSF, IL-15 and IL-27 (all at 48 h p.i.) (**Figures 4A, B**, **Table S5**). Thus, systemic injection of INSL5 confirmed the possible insulinotropic effects of INSL5, and resulted in changes in 7 cytokines, predominantly those involved with mediating homeostatic responses, particularly those affecting macrophage proliferation.

In parallel to plasma analyses, the levels of IL-17, IL-10, TNFα, and IFN-γ were assessed in splenic CD4+ and CD8+ T-cells using FLOW cytometry 24 and 48 h following INSL5 injection. CD4+ T-cells had a higher proportion of IL-17+ populations than CD8+ cells (**Supplementary File 4**), and IL-17 was decreased in CD4+ T-cells at 24 h (**Figure 4D**), replicating the alterations measured in plasma (**Figure 4A**). Anti-inflammatory IL-10 exhibited an increased trend in CD8+ T-cells, though not statistically significant (**Figure 4D**). Pro-inflammatory TNFα and IFN-γ exhibited an overall decreasing trend in both subsets of T-cells (**Supplementary File 4**), though significant effects in comparison to control were not observed likely due to the limited time release of TNFα and IFN-γ.

### INSL5 Affects Cell Proliferation and Immune Markers in Immune Cell Subsets

**Figure 5:**
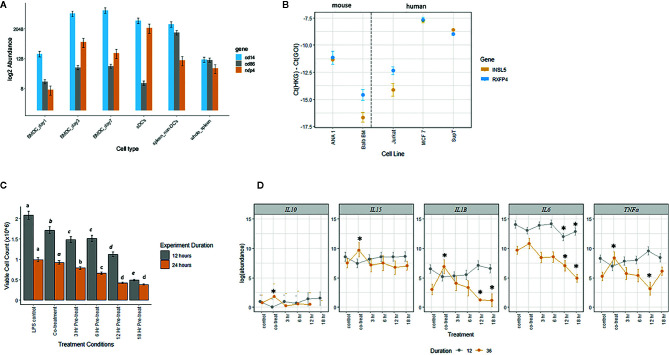
**(A)** Estimated abundance of *Rxfp4*, *cd86* and *cd14* in bone-marrow derived dendritic cells (BMDC’s) one, three, and seven days post-differentiation in GM-CSF, whole spleen, splenic dendritic cell (sDC’s), and non-dendritic cell (non-DC) fraction of spleen. **(B)** Expression of *Insl5* (yellow) and *Rxfp4* (blue) in two mouse macrophage lines, ANA-1 and Balb-BM, relative to the geometric mean of two housekeeping genes, *ubc*-2 and *S18* (left panel) and in three human cancer cell lines, Jurkat, MCF-7 and SupT, relative to *gapdh* and *rplpo*. **(C)**. Number of viable ANA-1 cells after 12 or 24 h stimulation with LPS in six treatment lines: LPS control, co-treatment with INSL5 and LPS, and pre-incubation in INSL5 for 3, 6, 12, or 18 h prior to LPS stimulation. Differences in the number of cells per treatment was assessed using ANOVA and treatments with significantly different means within an experiment (12 or 24 h) are indicated in letters. **(D)** Bayesian generalized linear mixed model analyses of the change in abundance of cytokines in six treatments following 12 or 36 h or LPS stimulation in ANA-1 cells. The log2 fold-difference between each treatment against LPS control was calculated using a standard z-test and then two-tailed p-values calculated. Only those comparisons that were significant after correcting for multiple tests (p,0.00067) were deemed significant. See methods for details, and **Table S7** for fold-change and p-values.

Given the broad expression of *Rxfp4* across immune tissues and cell subsets, we hypothesized that INSL5 may convey signals through major immune cell subsets, such as T-cells and dendritic cells. In agreement with the *in silico* data, dendritic cells and mouse cell lines derived from DCs (e.g. ANA-1 and Balb/c macrophages cell lines) expressed detectable levels of *Rxfp4* (**Figure 5A**); and ANA-1 cells also expressed *Insl5* (**Figure 5B**). *Rxfp4* expression increased over time in BMDC’s, and was higher in whole spleen (9-fold) than the non-DC’s portion of spleen (**Figure 5A**). No *Rxfp4* expression was detected in splenic T-cells (not shown). *Rxfp4* was expressed in all three human cancer cell lines, Jurkat, MCF-7 and SupT, while *Insl5* was also expressed in the latter two, with both genes showing the highest expression in the breast cancer MCF-7 cells (**Figure 5B**). We then used mouse macrophage ANA-1 cells to examine the effect of INSL5 on cytokine expression following inflammatory activation of cells using LPS. Overall, INSL5 appeared to suppress cell growth; the number of viable cells was significantly higher in the LPS control than those co-treated with INSL5/LPS following 12 h of stimulation (**Figure 5C**).

Secondly, for all cells pre-treated with INSL5, the number of viable cells after both 12 and 24 h was significantly lower than the control or co-treatment lines (**Figure 5C**). For cytokine expression, incubation of ANA-1 cells in INSL5 resulted in no significant differences in expression of IL-10 or IL-5 (not shown as levels were zero for many time points). Levels of IL-15 were significantly higher in the co-treatment and 3 h INSL5 pre-treatment compared to control for cells stimulated for 36 but not 12 h, which is consistent with the *in vivo* injection experiment. Levels of IL-1β did not change significantly among treatments following 12 h of stimulation, however co-treatment of cells with INSL and LPS resulted in higher expression of IL-1β after 36 h, and cells pre-treated with INSL5 for 12 or 18 h exhibited significantly lower levels of pro-inflammatory cytokine IL-1β (**Figure 5D**). Similarly, levels of the pro-inflammatory cytokine IL-6 were significantly lower following INSL5-LPS co-treatment than in the control, and lower in cells pre-treated with INSL5 for 12 or 18 h prior to LPS stimulation for both time courses (**Figure 5D**). Finally, levels of TNFα were not different among treatments following 12 h of stimulation, but they were higher in cells co-treated with INSL5/LPS after 36 h of stimulation with LPS, and lower than control cells in cells pre-treated with INSL5 for 12 h (**Figure 5D**) (see **Table S7** for all results). Collectively, this suggest that INSL5 has an overall inhibitory effect on ANA-1 macrophage proliferation and an anti-inflammatory effect on cytokine release.

## Discussion

Using a combination of *in silico* and experimental approaches, we find evidence that INSL5 can alter cytokine profiles in the peripheral immune system potentially *via* its cognate receptor, RXFP4, which is expressed in both central and peripheral immune tissues, and on some immune system subsets, particularly those involved with the innate immune system. We detected expression of *Insl5* in thymus and colon; and find that in thymus, *Insl5* was most highly expressed in the youngest cohort of mice, where it was strongly correlated with expression of *Il-7*, a marker of early thymocyte development. Through data mining, we show that *Insl5* is specifically expressed in some immune cell subsets, particularly in the thymus (DP and DN thymocytes, cTEC’s), intestinal epithelial lymphocytes (IEL’s) and a few CD4+ T-cell subsets. Through phylogenetic footprinting we identified strong evidence for regulation by immune-related TF’s including: STAT’s (STAT 1, STAT 4, STAT 5a/5b), which work *via* JAK/STAT signaling pathways to regulate the expression of genes in cell survival, proliferation, immune response, and hematopoiesis; FOXP3, a master regulator of the development of T-cells, as well as TCF-1 and NF-AT, which enhance thymus-specific gene expression (**Supplementary File 2**).

For RXFP4, using qPCR, we found higher expression of *Rxfp4* than *Insl5* across all immune tissues (except colon), including the thymus. Our experimental work in thymus was based on examination of whole thymic tissue using verified Taq-man probes, but our finding of higher expression of *Rxfp4* than *Insl5* in thymus runs contrary to our bioinformatics data mining which identified high expression of *Insl5* in many thymus-specific RNA-sequencing datasets at NCBI (**Table S2**). One possible explanation for this result is that *Rxfp4* is expressed at lower levels in many thymic cell subsets, while *Insl5* may be expressed at higher levels in a few cell subsets (e.g. DP thymocytes), a hypothesis that is consistent with our database mining. We further find that the expression of thymic *Rxfp4* was correlated with that of *FoxN1* and *Ghr* across all age cohorts, and that *Rxfp4* expression was not lower in the oldest cohort of mice. FoxN1 is a transcription factor that regulates hundreds of genes in the thymus during T-cell development, particularly contributing to the function of thymic epithelial cells (TEC’s) (31, 37), while GHR (via GH secretagogues) is also associated with maintenance of TEC’s and thymus cytoarchitecture (29, 30). Collectively, these results indicate that further investigation into the potential role of INSL5 in thymocyte development, perhaps during DN-DP selection or through the development FOXP3+ T-regulatory cells is warranted.

The interplay between endocrine hormones and the immune system is complex. However, many appetite regulating hormones can also regulate levels of cytokines *in vitro* and systemically, markedly GLP-1 (38–40). INSL5 and GLP-1 are co-secreted from L-cells together with PYY (41); notably all three of these gut hormones were found to be upregulated following i.p. injection of INSL5, consistent with recent transcriptomic data showing that they are co-regulated (41). GLP-1 is emerging as a player not only in metabolic glucose control, but also in orchestrating immune responses in the gut (40). Since INSL5 levels were shown to be highly elevated in the distal guts of germ-free mice (42), we reasoned that their response to changing microbial conditions in the gut (7) could mediate an anti-inflammatory response to facilitate microbial colonization.

Thus, we performed i.p. injections in a mouse model using concentrations of INSL5 peptide found to have significant effects on the feeding response by (9) and looked for changes in plasma cytokines which are indicative of a systemic immune response. We aimed to validate the concentration and mode of INSL5 injection by confirming the insulinotropic effect following i.p. injection of INSL5 and measured significant increases in GLP-1 (6 and 24 hr, p.i.), Insulin and C-peptide (24 hr, p.i.) after injection as well as a transient increase in PP followed by PYY, when compared to sham injection. Although this suggests an overall insulinotropic effect of INSL5 as found in other research (6, 8), levels of glucagon, which impedes insulin secretion, were also higher, underscoring the need for more research on the effect of INSL5 in glucose metabolism as suggested by other authors (3, 7).

We then sought to assay levels of a discovery panel of cytokines representing cytokines with diverse roles in immune cell signaling 6, 24, and 48 h post injection. Overall, 7 cytokines exhibited changes in expression level at one or more time points following injection, three of them were chemokines or homeostatic cytokines that specifically modulate macrophage proliferation (MIP-2, G-CSF and M-CSF) (43, 44) or other homeostatic processes (IL-7 and IL-15) (45–47) while the other two fell into regulatory (IL-27) or Th1/Th2 responses (IL-5) (48) In a parallel experiment, we measured a decrease in inducer of inflammation IL-17 in the CD4+ splenic T-cells isolated from INSL5 injected mice (49), as well as a marginal increase in the anti-inflammatory IL-10 in both CD4+ and CD8+ T-cells, more notably in the latter (50).

Next, we wanted to further probe whether specific immune cell subsets expressed *Rxfp4*, and then responded to INSL5 following LPS stimulation. We show here, for the first time, that naïve BMDCs differentiated in the presence of GM-CSF, do not express *Rxfp4* on the first day of differentiation, but do on days 3 and 7 of differentiation. Further, expression of *Rxfp4* was six-fold higher in the sDCs compared to the non-DCs fraction. Turning to mouse and human cell lines, we found that *Rxfp4* was expressed in both mouse macrophage cell lines (ANA-1 and BALB/c), as well as three human cancer cell lines (Jurkat, MCF-7, and SupT). To test the hypothesis that an *Rxfp4*-expressing macrophage cell line may mediate immune-responses to INSL5, we employed the mouse BMDM cell line, ANA-1, and conducted an experiment to test whether cells pre-incubated with INSL5 had altered levels of cytokines relative to cells not pre-incubated with INSL5 (co-treatment) or to the control (LPS alone). All treatments were stimulated in LPS for two-time courses (12 or 36 h) and both viable cell counts and levels of six cytokines assayed by qPCR. We chose cytokines that were observed to show systemic effects in the injection experiment (IL-5 and IL-15), or are known to be important immune modulators (IL-10, IL-1B, IL-6 and TNFα). Several of the cytokines showed elevated levels of expression in the co-treatment compared to control (IL-10, IL-15, IL-1β, and TNFα) after 36 h of LPS stimulation, and both IL-1β and IL6 showed a significant reduction in expression when cells were pre-incubated in INSL5 for 12 or 18 h following both 12 and 36 h of stimulation (IL-6) or following only 36 h of stimulation (IL-1β).

These results were also consistent with the injection experiment for IL-15 and IL-6 (IL-1β levels were not high enough for detection in the *in vivo* experiment) and suggest that INSL5 could alter the inflammatory response under some conditions. Pro-inflammatory IL-6 is secreted in response to microbial proteins and is elevated in gut inflammatory diseases (51). Tessaro et al. (52) found that stimulation of BMDM’s with insulin and LPS led to a significant increase in IL-6 and TNFα, *via* upregulation of the PI3K and ERK 1/2 pathways, in diabetic but not non-diabetic mice, but found a downregulation of IL-6 and TNFα following insulin + LPS in tissue resident macrophages (alveolar and peritoneal macrophages) (52). Here, we find evidence of a down-regulation of IL-6 and IL-1β and to a lesser extent in TNFα in the BMDM’s from a non-diabetic C57/Bl6+ mice. The findings that incubation in INSL5 depressed proliferation of ANA-1 cells, and reduced pro-inflammatory cytokine release, suggest that the effect of INSL5 on tissue-resident and non-resident macrophages and dendritic cells warrants further exploration.

Our data mining uncovered broad similarities in the expression of *Insl5/Rxfp4* in human and mouse when data on both species was available, but there are known differences between the species in both the tissues and cell subsets that express them, even in colon (53). Of relevance here, most of our experimental and data mining results for the expression of *Insl5/Rxfp4* in thymus are derived from mouse, because no human data was available. In the gut, however, there is evidence that *Rxfp4* is expressed in immune-relevant cell types in both human and mouse, including goblet cells (53), the vagus nerve in mouse (9), as well as splenic DC’s (here). Thus, it is possible that mouse is a reasonable model to study the possible immune function of these genes, despite the fact that the peptide sequence conservation between the species is modest (75% for INSL5 and 74% for RXFP4), and there are genomic re-arrangements in the regions housing the genes, a situation potentially associated with the pseudogenization of the genes in some murids.

If INSL5 has immunomodulatory effects in the gut-immune axis, it would be expected to play a role in metabolic syndromes, such as inflammatory bowel disease and Crohn’s. Retrieval of the RNA-seq data at the EMBL-EBI expression atlas (www.ebi.ac.uk/gxa/home), last accessed May 2019) uncovered 26 experiments for *Insl5* and 25 for *Rxfp4* in which significant differences were observed between treatment arms: five of these studies found decreased expression of *Insl5* or *Rxfp4* in association with metabolic disorders such as Crohn’s disease, while 22 (11-*Insl5*, 11-*Rxfp4*) found significant changes in gene expression associated with various cancer including colon and breast (summarized in **Table S3**). Further, a clinical study in humans undergoing bariatric surgery found that plasma levels of INSL5 were inversely correlated with C-reactive protein (CRP), a marker of inflammation secreted by the liver into plasma in response to acute stress (16), and positively correlated with BMI and insulin resistance (11, 16). Taken together with the results presented here, we propose that INSL5 and RXFP4 play roles in immune system signaling, both in the central and peripheral immune systems. Our findings indicate potential roles in thymocyte selection in the central immune system as well as on macrophage proliferation in the peripheral immune system. Further investigation is required to tease apart the specific roles of INSL5-RXFP4 and aid our understanding of the peptide’s role in the immune-metabolic axis and conditions of health and disease.

## Data Availability Statement

Publicly available datasets were analyzed in this study. Existing datasets are available in a publicly accessible repository (SRA and EMBO/EBI). The relevant accession numbers can be found in our **Supplementary Files 1, 2**. For cytokine and metabolic assays, and log 2 fold changes, p-values used for the qPCR data, the raw values can be found in **Supplementary File 3**.

## Ethics Statement

The animal study was reviewed and approved by Animal Care Committees of University of Winnipeg and University of Manitoba.

## Author Contributions

SG and BV conceptualized the experimental work in collaboration with JU, SY, and AS. Experiments were performed by BV, CO, JD, and DR. SG, BV, CO, and SY analyzed the data. SG and BV wrote the manuscript with editorial contributions from co-authors. All authors contributed to the article and approved the submitted version.

## Funding

This research was funded by a Discovery Grant DG-06203 from the Natural Sciences and Engineering Research Council of Canada to SG, an NSERC-USRA to JD, a University of Winnipeg Graduate Student Scholarship to BV, and a University of Winnipeg Major Grant, awarded to SG.

## Conflict of Interest

The authors declare that the research was conducted in the absence of any commercial or financial relationships that could be construed as a potential conflict of interest.
