# Supplementary material for: Immune System Effects of Insulin-Like Peptide 5 in a Mouse Model
Source: Front Endocrinol (Lausanne). 2021 Jan 14;11:610672. doi: 10.3389/fendo.2020.610672 (PMC7841425; doi:10.3389/fendo.2020.610672)
Supplement: Supplementary file 1 [file DataSheet_1.zip › 610672_Supplementary/Supplementary_File_2.PDF]

Immune System Effects of Insulin-like Peptide 5 in a Mouse Model.

TableS2a Reported functions of transcription factors identified in the study, and their classification based on the ENCODE project.

**Transcription Factor**	**Reported Literature Functions**	**Encode Classification**
COE1 (Ebf1)	Involved in CNS functioning and neurogenesis and neuronal differentiation. Secondary roles include morphological differentiation and immune specialization.	Developmental Process
HES-1	Involved in neural, cardiovascular, and digestive developments, as well as cellular differentiation.	Developmental Process
INSAF (Insulin activating factor)	Activates ICE in insulin expressing cells but not in non-insulin expressing cells. ICE in turn regulates pancreatic B-cell-type-specific-transcription resulting in insulin transcription.	Metabolic Process
MEF2	Involved in skeletal, smooth and cardiac muscle development by inducing myogenesis and differentiation of myocytes. Also plays a role in cell proliferation.	Developmental Process
MyoD	Plays a role in myoblast formation, proliferation and *specification* in skeletal muscle. Regulates hyperplasia and hypertrophy of skeletal muscle.	Cellular Process
Myogenin	Involved in myocytes and myoblasts development. Required for muscle *differentiation*.	Developmental Process

**Transcription Factor**	**Reported Literature Functions**	**Encode Classification**
PTF-1	Inhibition of neuronal function in the spinal cord, cerebellum and retina. Responsible for GABAergic neuron production in spinal cord and cerebellum.	**Response to stimulus**
USF-1 (Upstream Transcription Factor)	Involved in embryonic development, induction of cell cycle arrest (Cdk4 suppression) and DNA repair pathway (NER) in UV-induced DNA damage, and promotion of immune response by expressing Ig light chain genes to provide tissue/cell protection and tumor suppression	

**Transcription Factor**	**Reported Literature Functions**	**Encode Classification**
AP-1	Differentiation of naïve T cells to T_H1/T_H2, regulation of cytokine genes with NFAT, Jun/AP-	Immune System Process

	1 controller of cell proliferation. Tumor suppression and angiogenesis	
ATF-1	Implicated as part of a signal transduction pathway responding to UV-induced DNA damage	Cellular Process
ATF-2	Involved in stress-induced epigenome changes and DNA damage response; also involved in the CNS and skeletal development	Cellular Process
ATF-3	Initiates broad cellular stress response to a wide variety of signals, including those initiated by cytokines, genotoxic agents or physiological stress	Cellular Process
c-Fos	Involved in embryonic development (organogenesis: brain, bone and cartilage) and regulation of cell growth and proliferation	Developmental Process

**Transcription Factor**	**Reported Literature Functions**	**Encode Classification**
c-Jun	Involved in embryonic development (embryonic programmed cell death, PCD, and organogenesis), control of cell cycle (G1 to S phase) and proliferation, mitigation of cellular stress and apoptotic responses when exposed to genotoxic agents	Developmental Process
C/EBP (CCAAT/ enhancer binding protein)	Regulates energy metabolism, cellular growth and differentiation (adipogenesis, hematopoiesis and osteoclastogenesis), liver regeneration, participates in inflammatory and immune responses, and synaptic plasticity underlying memory formation	
CREB (cAMP response element-binding protein)	Cellular stress response. Regulator of cellular proliferation, differentiation (e.g., myeloid cells and megakaryocyte) and survival, including a role in hematopoiesis. Implicated in glucose homeostasis and learning and memory. It also plays important role in physiology of pituitary gland, in regulating spermatogenesis, and in the response to circadian rhythms	
DBP	Regulator of circadian rhythm in the brain	Response to Stimulus

Transcription Factor	Reported Literature Functions	Encode Classification
ENKTF1	Regulates neuropeptide inducible transcription and upregulates cAMP levels in the presence of a functional ENKCRE-2 element	Response to Stimulus
Fra-1 (Fos-related antigen 1)	Regulates gene expressions involved in cell growth, apoptosis, and cell motility, and genes involved in tissue/cell remodeling including MMP-1/-2/-9. Confers pulmonary protection by maintaining cellular homeostasis.	Cellular Process
JunB	Regulation of T-helper cell differentiation. Also implicated in myeloid cell and Th2 cell differentiation	Immune System Process
JunD	Protection of fibroblasts from p53 induced apoptosis and cell senescence. Regulates lymphocyte proliferation	Immune
Nrf2/Mafk	Protection from oxidative stress. Involved in the regulation of antioxidant-defense through the transcriptional activation of antioxidant response elements (AREs) in response to oxidative/electrophile stress.	Cellular (Immune?)

Transcription Factor	Reported Literature Functions	Encode Classification
XBP-1	Required for differentiation of hepatocytes and plasma cells. Involved in the transition of B lymphocytes to plasma cells. Also involved in EC proliferation, autophagy and apoptosis. A signal transducer in the ER.	
**Transcription Factor**	**Reported Literature Functions**	**Encode Classification**
Cdx2 (Cdx3)	Regulates gastrulation including mesoderm development. Also plays a role in intestine development.	Developmental Process
Crx	Implicated in the differentiation of photoreceptor cells (rods and cones). Expressed in both embryonic and adult photoreceptors.	Cellular Process
HOX	Regulation of embryo morphological body patterning, especially the anterior-posterior (AP) axis and segmentation (i.e.,	Developmental Process

	cell growth, differentiation, apoptosis and movement)	
Cutl1 (cut-like 1; aka Cux/CDP)	Regulator of cellular proliferation, motility and cell cycle progression (late G1 and the S phase)	Cellular Process

**Transcription Factor**	**Reported Literature Functions**	**Encode Classification**
Nkx2.1	Involved in the development of the thyroid, lungs and ventral forebrain. Also plays a role in the development of the neural crest, GABA and cholingernic neuron specification, and development of the medial ganglionic eminence (MGE) in the forebrain	Developmental Process
Nkx6.2	Involved in pancreatic development, specifically the differentiation of alpha and beta cells	Developmental Process
Pdx1 (IPF1)	Regulates pancreatic development and pancreatic islet cell functions. Reduced pdx1 impairs glucose-stimulated insulin secretion	Developmental Process
**Transcription Factor**	**Reported Literature Functions**	**Encode Classification**
POU1F1 (Pituitary-specific POU-homeodomain; aka Pit1)	Regulates the development of the anterior pituitary. Also regulates the expression of three hormones (GH, PRL, and TSH-beta) thus serving an essential function in the differentiation/proliferation of somatotropes, lactotropes and thyrotropes	Developmental Process
POU2F1 (aka OCT-1)	Promotes resistance to oxidative and genotoxic stress, serving as a stress response effector	Cellular (Immune?)

**Transcription Factor**	**Reported Literature Functions**	**Encode Classification**
HNF-1 (Hepatocyte nuclear factor 1)	Embryonic pancreas development and maintenance of adult islets of β cells, organogenesis (neural tube, lungs and genital tracts) and regulation of hepatic genes involved in detoxification and energy (glucose, amino acids and lipid) metabolism	Developmental Process/metabolic process
**Transcription**	**Reported**	**Encode**

Factor	Literature Functions	Classification
NF-I (Nuclear factor I)	Regulation of pre and postnatal organogenesis (brain, blood, testis, liver, thyroid and pituitary) and development (adipogenesis), postnatal mammary gland development and lactation, and cellular DNA replication and growth	Developmental/ Cellular

Transcription Factor	Reported Literature Functions	Encode Classification
HNF3-α/-β/-γ (FoxA1/A2/A3)	Organogenesis (liver, intestine, and pancreatic islet cells and acinar cells), glucose homeostasis and hormone synthesis and regulation of metabolism	Developmental/ metabolic
FOXP3 (Forkhead box P3)	Regulates the development, stability and function of Treg cells (CD4+CD25+ Treg cells; it can also transiently expressed in activated CD4+CD25- Teff cells)	Immune

Transcription Factor	Reported Literature Functions	Encode Classification
MF (FOXB1 or fkh5)	Highly expressed in the developing hypothalamus and thalamus and regions of the spinal cord (CNS); regulates lactation by forming lobuoalveolar development	Developmental

Transcription Factor	Reported Literature Functions	Encode Classification
c-Ets-1/-2 (E twenty-six transformation-specific)	Promotion of embryo vascularization (first trimester), cell growth and differentiation during embryogenesis (endothelial cells), induction of angiogenesis and wound healing, and regulation of cellular stress in response to oxidative agents	Developmental Cellular
Fli-1 (Friend murine leukemia virus-induced erythroleukemia-1)	One of ten key TFs that control hematopoietic stem/progenitor cell maintenance and differentiation. Regulates differentiation of T & B cells (for B cells, it controls proliferation and survival) and the production of cytokine and chemokine production. It also controls angiogenesis by being expressed in vascular endothelial cells	Cellular/Immune
PEA3 (Polyomavirus	Encodes matrix-degrading proteolytic enzymes that are involved in extracellular matrix degradation to permit tissue	Cellular/ Developmental

enhancer activator 3 homolog; aka ETV4 or E1Af)	rearrangement. Regulate the transcription of several proteinases involved in tissue remodeling, including the matrix metalloproteinases (MMP) and collagenases-IV/gelatinase B (MMP-9). Experimental evidence indicates that members of PEA3 subfamily to be involved in CNS/PNS nervous system development	
**Transcription Factor**	**Reported Literature Functions**	**Encode Classification**
PU.1 (Purine-rich box 1)	Regulation of fetal hematopoiesis (macrophage/B cell/early T cell differentiation and maintenance of long-term hematopoietic stem cell viability)	Immune
**Transcription Factor**	**Reported Literature Functions**	**Encode Classification**
ER (Estrogen receptor)	Regulates mammary gland (specifically ductal elongation) morphogenesis; preliminary experimental results suggesting its involvement in murine embryonic lung development and neuronal survival as early as embryonic day 15 (both for ER beta)	Developmental
PR (Progesterone receptor, aka Pgr or Prl)	Required for mammary gland development (e.g., alveologenesis and side branching formation during pregnancy to create lactation-competent mammary glands)	Developmental Process
VDR (Vitamin D receptor)	Regulates the differentiation of mesenchymal stem cells to fat and, chiefly, bone cells and bone formation.	Cellular
YY1 (Yin Yang 1)	Embryonic development, hematopoiesis, regulation of cell cycle control and differentiation and activation of DNA repair pathway	Developmental Process/Cellular

Transcription Factor	Reported Literature Functions	Encode Classification
GATA	Embryonic development, hematopoietic progenitor cell proliferation and development, T-cell differentiation and hormone synthesis	Developmental Process/Cellular
GR (Glucocorticoid	Embryonic development, glucose homeostasis, cellular stress, inflammatory and immune response,	Cellular

Receptor)	regulation cell of differentiation and survival, bone turnover and lung maturation	
SF1	Development of prenatal reproductive organs and network and maintenance of postnatal endocrine homeostasis	Developmental
RXR (Retinoid X Receptor)	Embryonic development (organogenesis and body patterning), regulation of cell proliferation, differentiation and survival, and modulation of immune and anti-inflammatory response	Developmental Process/Immune
T3R (Thyroid hormone – 3,5,3'-triiodothyronine – receptor; aka TR)	Profound effects on oxygen consumption and metabolic rate. Specifically, TH regulates the normal development of bone and bone growth, brown and white adipose tissues, pituitary hormone synthesis (e.g., GH), and in utero brain development during the neonatal period. In liver, TH stimulates enzymes regulating lipogenesis and lipolysis as well as oxidative processes (action of TH is exerted by T3R)	Developmental Process

Transcription Factor	Reported Literature Functions	Encode Classification
IRF1	Regulator of innate immunity: required for Th1 T-cell differentiation. Suppresses oncogenesis by inducing apoptosis.	Immune
LEF1/TCF	Involved in lymphocyte differentiation and expression. Also mediates Wnt signaling leading to cell proliferation.	Cellular
HMGI(Y)	Architectural transcription factor with a role in tumorigenesis.	
WT1	Plays a role in urogenital development (including sex differentiation) and Wilms tumor suppression. Possible oncogene.	Developmental Process
CP2	Regulator of hemoglobin switching (a-globin promoter) and male development.	Reproductive
c-Myb (V-Myb avian myeloblastosis viral oncogene homolog)	Regulation of hematopoietic stem cell (erythrocyte, megakaryocyte and T cells) proliferation, survival and differentiation	Cellular/ Immune

Transcription Factor	Reported Literature Functions	Encode Classification
SRF	Involved in smooth and cardiac (and skeletal?) muscle development including proliferation and differentiation.	Developmental Process
PUR alpha/beta	Involved in brain development and plasticity (specifically in hypothalamus), found in GnRH neurons. Plays roles in cell cycle regulation.	Developmental Process
NF-kappaB	Regulation of hematopoiesis. Involved in the innate and adaptive immune response, induction of cytokines.	Immune
E2F-1	Activator of the cell cycle, specifically the transition to S phase and inhibition of quiescence.	Cellular
AML1 (Runx1)	Involved in hematopoiesis (liver, spleen, kidney, thymus) and later-stage T lymphocyte differentiation.	Developmental Process
AP-2alpha (Tfap2a)	Implicated in neural crest and craniofacial development.	Developmental Process
p53	Tumor suppressor – induces cell arrest and apoptosis. A component of the DNA-damage pathway.	Cellular
Pax5	Involved in hematopoiesis of B-cells. Controls B-cell fate and lineage commitment.	Celllular
NF-AT (Nuclear factor of activated T cells)	Regulation of thymocyte activation and differentiation (T-helper 1 and 2) and cytokine (IL-2/-4/-10, IFN-γ) production	Immune

Transcription Factor	Reported Literature Functions	Encode Classification
STAT1/4/5 (Signal Transducers and Activators of Transcription 4/5)	Regulation of immune responses (production of IL-4, IL-12, IFN-α, and IFN–γ; T helper 1 and 2 cell differentiation and T lymphocyte and NK cell survival), mammary gland development and lactogenesis (for STAT5a), cell cycle progression and apoptosis, and pituitary GH production and liver gene expression (for STAT5b)	Immune

VISTA PIP: Human vs Mouse INSL5

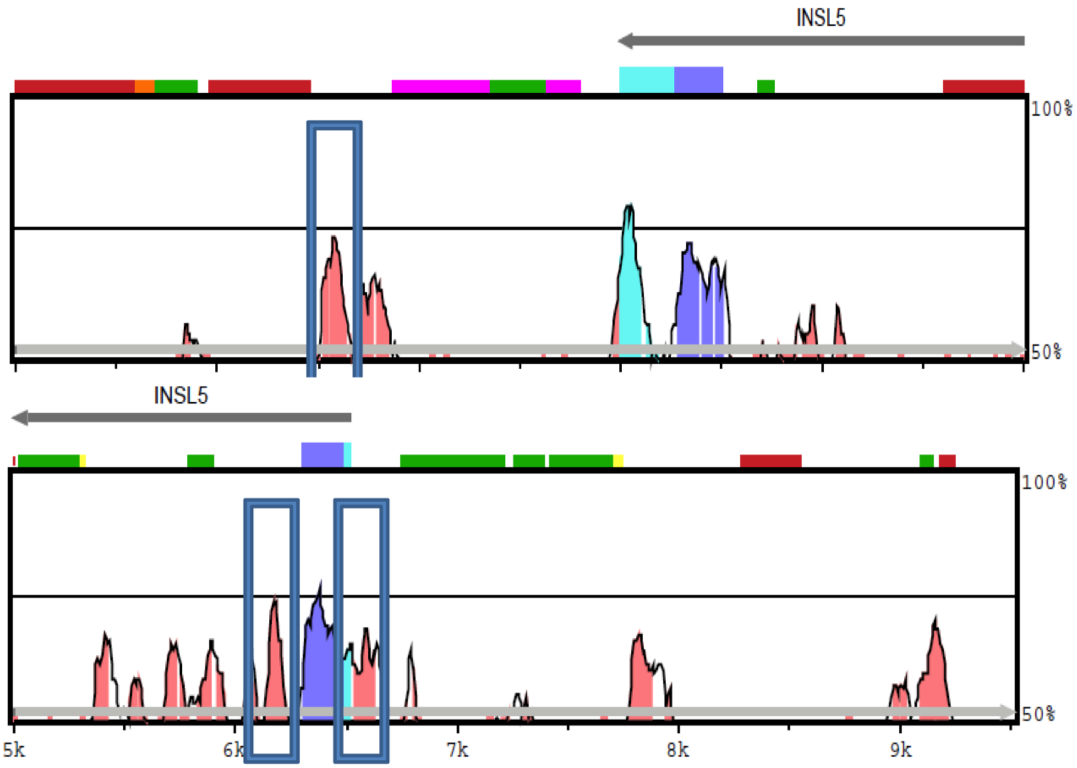

SuppFigure 2a mVISTA percent identity plot. Orthologous human and mouse *INSL5* genes and the 5' and 3' noncoding three thousand base pair sequences that potentially house gene regulatory elements were aligned using the Shuffle-LAGAN algorithm. The VISTA curve was generated by specifying 70.0% conservation threshold and a sliding window of 20-bp with human serving as the reference. The conserved noncoding sequences (CNS) are highlighted in pink and the conserved exons in blue. Any CNS peaks reaching or exceeding the set threshold were identified as candidate *cis*-regulatory modules, which were used to search for putative TFBS motifs. They are labeled in blue rectangles

VISTA PIP: Human and Mouse RXFP4

Human RXFP4

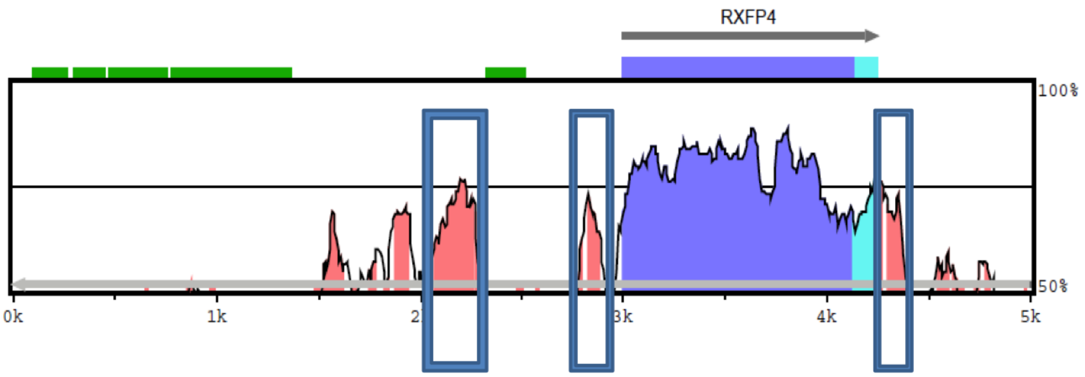

SuppFigure2b mVISTA percent identity plot. Orthologous human and mouse *RXFP4* genes and the 5' and 3' noncoding three thousand base pair sequences that potentially house gene regulatory elements were aligned using the Shuffle-LAGAN algorithm. The VISTA curve was generated by specifying 70.0% conservation threshold and a sliding window of 20-bp with human serving as the reference. The conserved noncoding sequences (CNS) are highlighted in pink and the conserved exons in blue. Any CNS peaks reaching or exceeding the set threshold were identified as candidate *cis*-regulatory modules, which were used to search for putative TFBS motifs. They are labeled in blue rectangles.

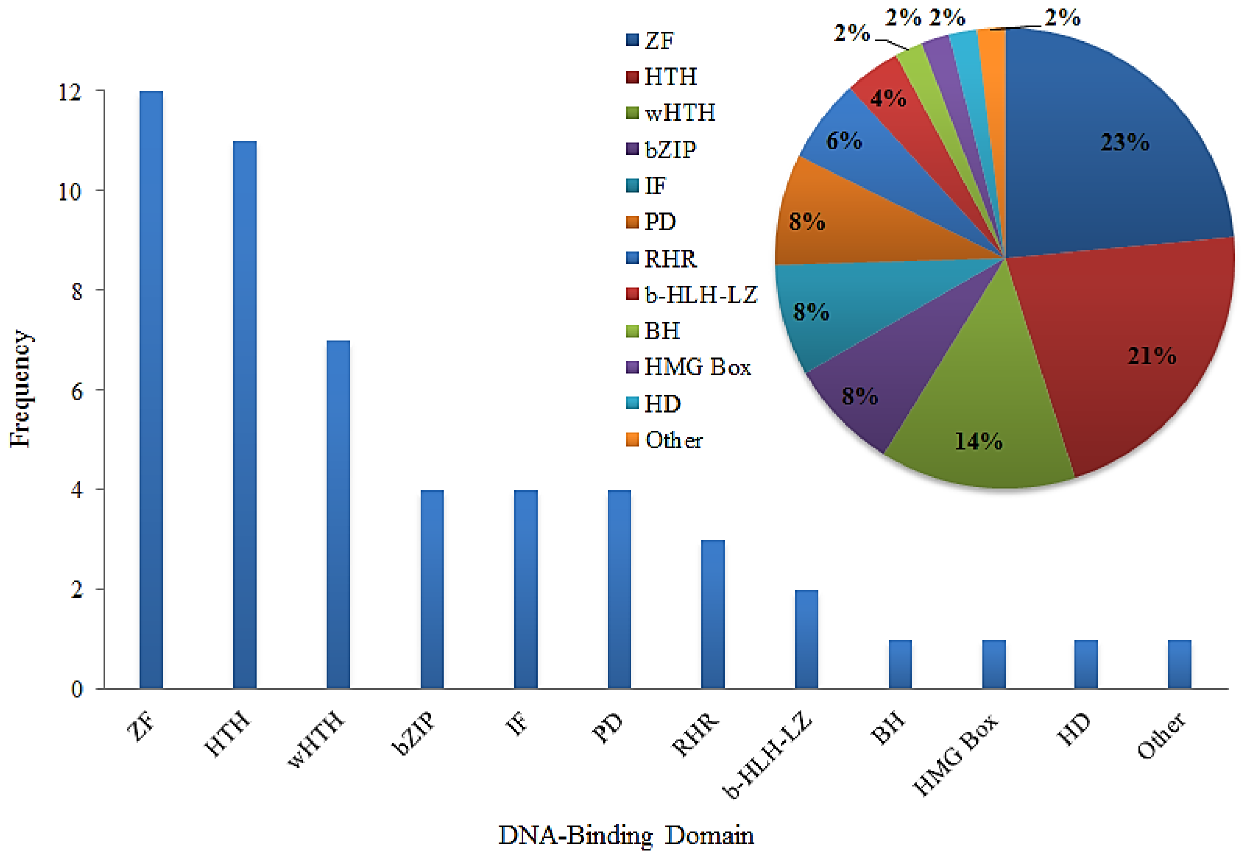

SuppFigure 2c. Classification of TFs based on DBDs. A total of 51 TFs were predicted to bind to the INSL5/RXFP4 candidate cis-regulatory elements by PROMO, with the zinc-finger, helix-turn-helix and winged helix-turn-helix motifs making up the three largest TF families. The pie chart associated with the histogram represents the TF DBD distributions in percentage values. ZF: zinc-finger; HTH: helix-turn-helix; wHTH: winged helix-turn-helix; bZIP: basic leucine zipper; IF: immunoglobulin fold; PD: paired domain; RHR: rel homology region; b-HLH-LZ: basic helix-loop-helix leucine zipper; BH: basic helix; HMG Box: high mobility group box; HD: homeodomain.

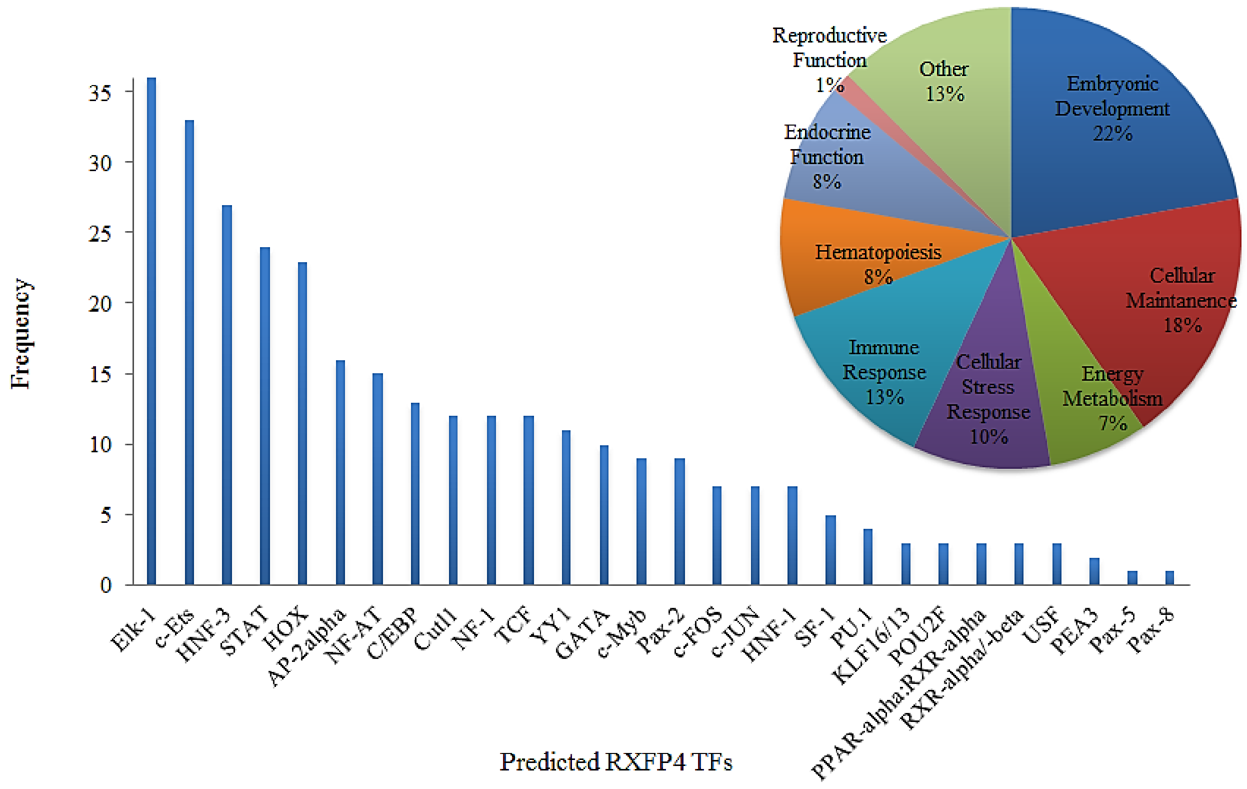

Supp Figure 2d. Predicted TFs that bind to the candidate *cis*-regulatory elements of the *RXFP4* gene. The biological roles of each TF were found by conducting literature searches and summarized using a functional chart. The accompanying pie graph shows the nine functional categories used with the percentage value associated with each category. Other:disease

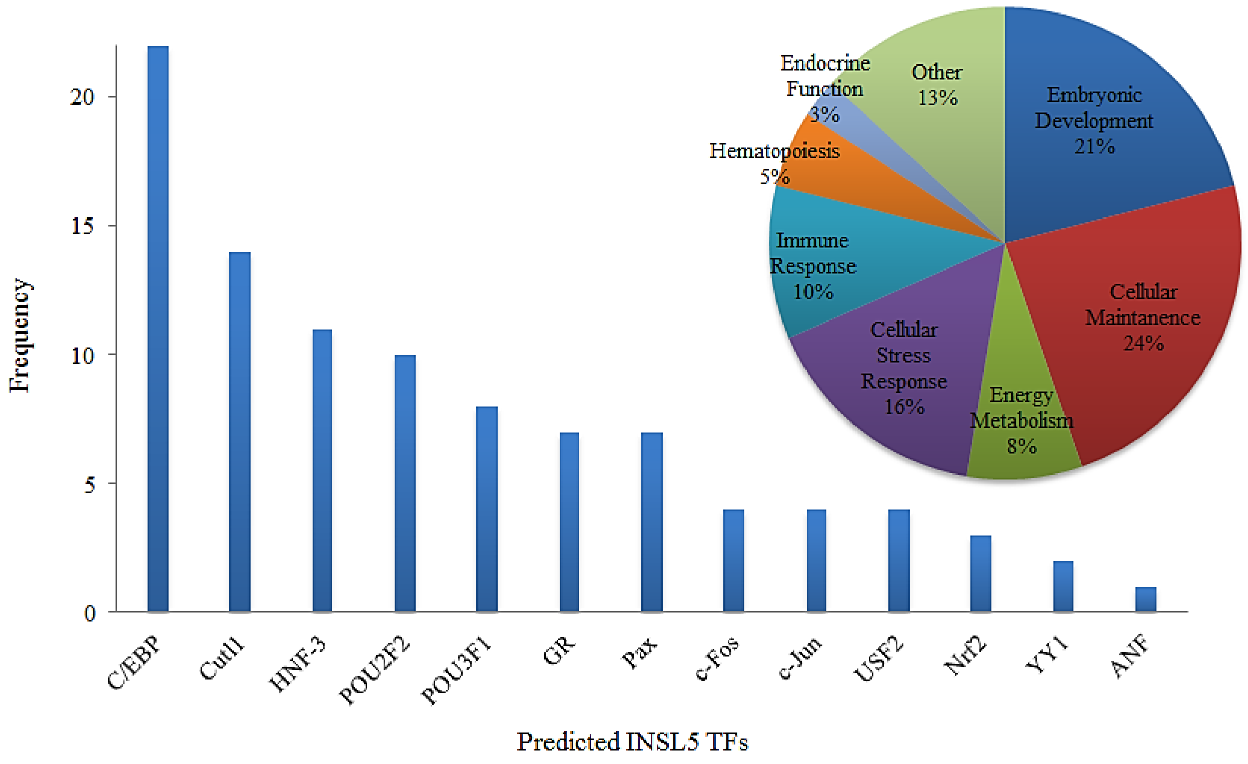

SuppFigure2e Predicted TFs that bind to the candidate *cis*-regulatory elements of the *INSL5* gene. The biological roles of each TF were found by conducting literature searches and summarized using a functional chart. The accompanying pie graph shows the nine functional categories used with the percentage value associated with each category.

SuppTable S2b – Transcription factors identified as potentially regulating the expression of Insl5 and/or Rxfp4 based on the classification by Vazquiera (2009) – as outline in SuppTableS2a above.

	Embryonic Development	Cellular Maintenance	Energy Metabolism	Cell Stress Response	Immune	Hematopoiesis	Endocrine	Reproductive Function
	AP-2 RXR-α	AP-2alpha	HNF-1	c-Ets	GATA1	c-Myb	HNF-3	STAT5a
	c-Ets	c-FOS	HNF-3	c-JUN	GATA3	GATA1	STAT5b	NF-1
	c-FOS	c-JUN	PPARα:RXRα	PPARα:RXRα	KLF16/13	KLF16/13	GATA1	
	c-JUN	Cutl1			NFAT1/2/3	Pu-1		
	GATA1	Elk-1			POU2F2	YY1		
	HNF-1	NF-1			Pu-1			
	HOX	STAT5b			RXR-α/β			
	NF-1	YY1			STAT1			
	Pax-2/5				STAT4			
	PEA3				TCF1			
	POU2F2				USF1/2b			
	RXR-α/β				RUNX3			
	USF1/2b				STAT5a/5b			

SuppFigure 2f: Potential binding sites of thymocyte specific TF's for INSL5: NFAT, TCF, FOXP3,STAT's, GR

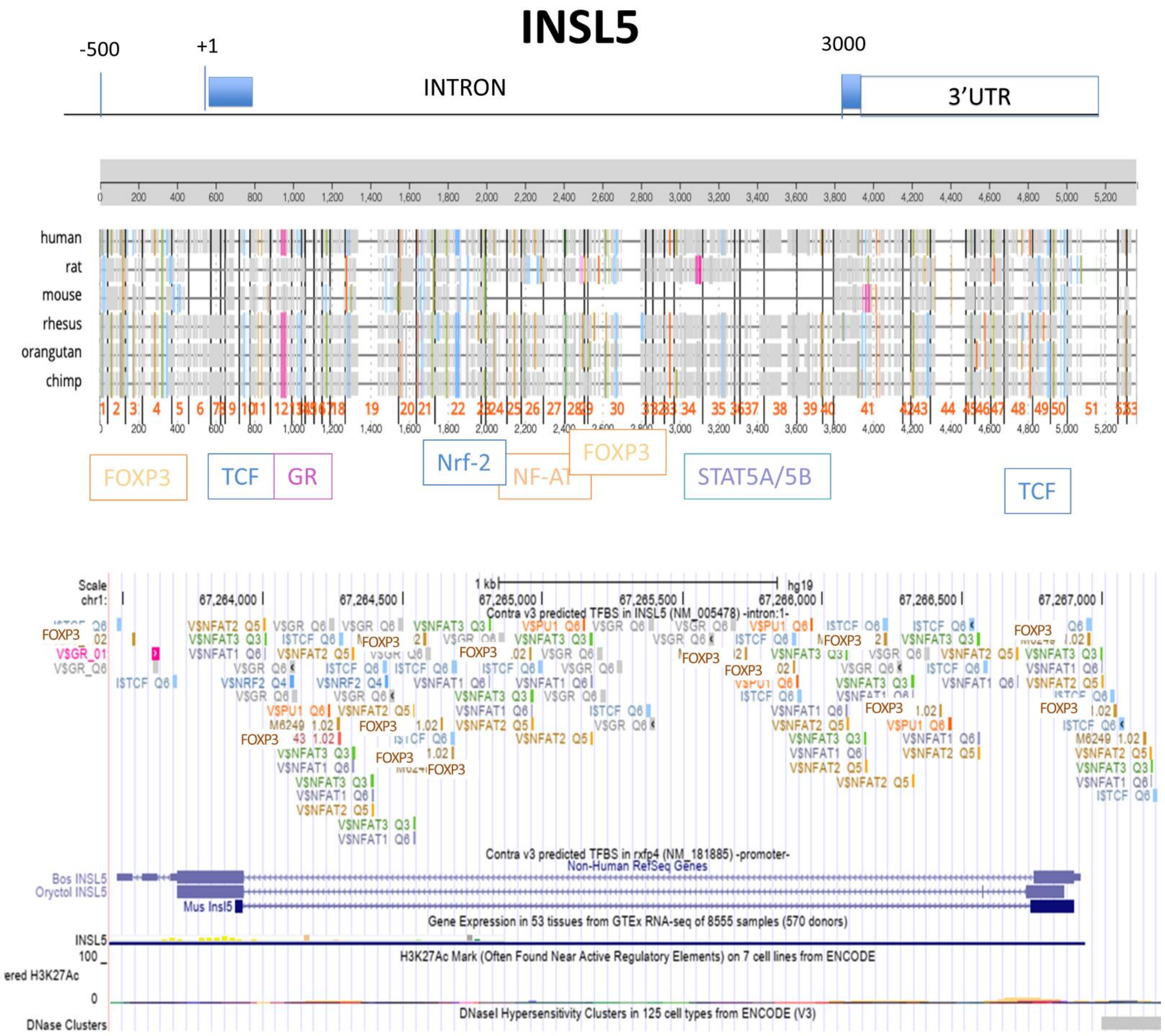

a

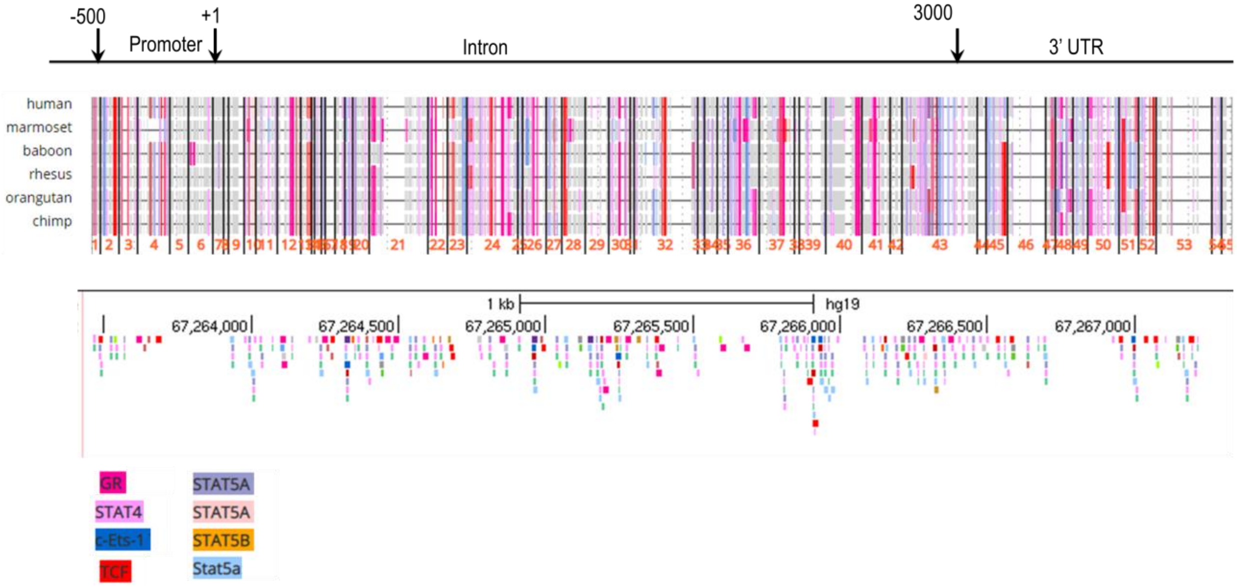

-500
Promoter
+1
Intron
3000
3' UTR
human
marmoset
baboon
rhesus
orangutan
chimp
1 kb
hg19
67,264,000
67,264,500
67,265,000
67,265,500
67,266,000
67,266,500
67,267,000
GR
STAT4
c-Ets-1
TCF
STAT5A
STAT5A
STAT5B
Stat5a

SuppFigureS2g: Potential and evidence for TFBS for RXFP4

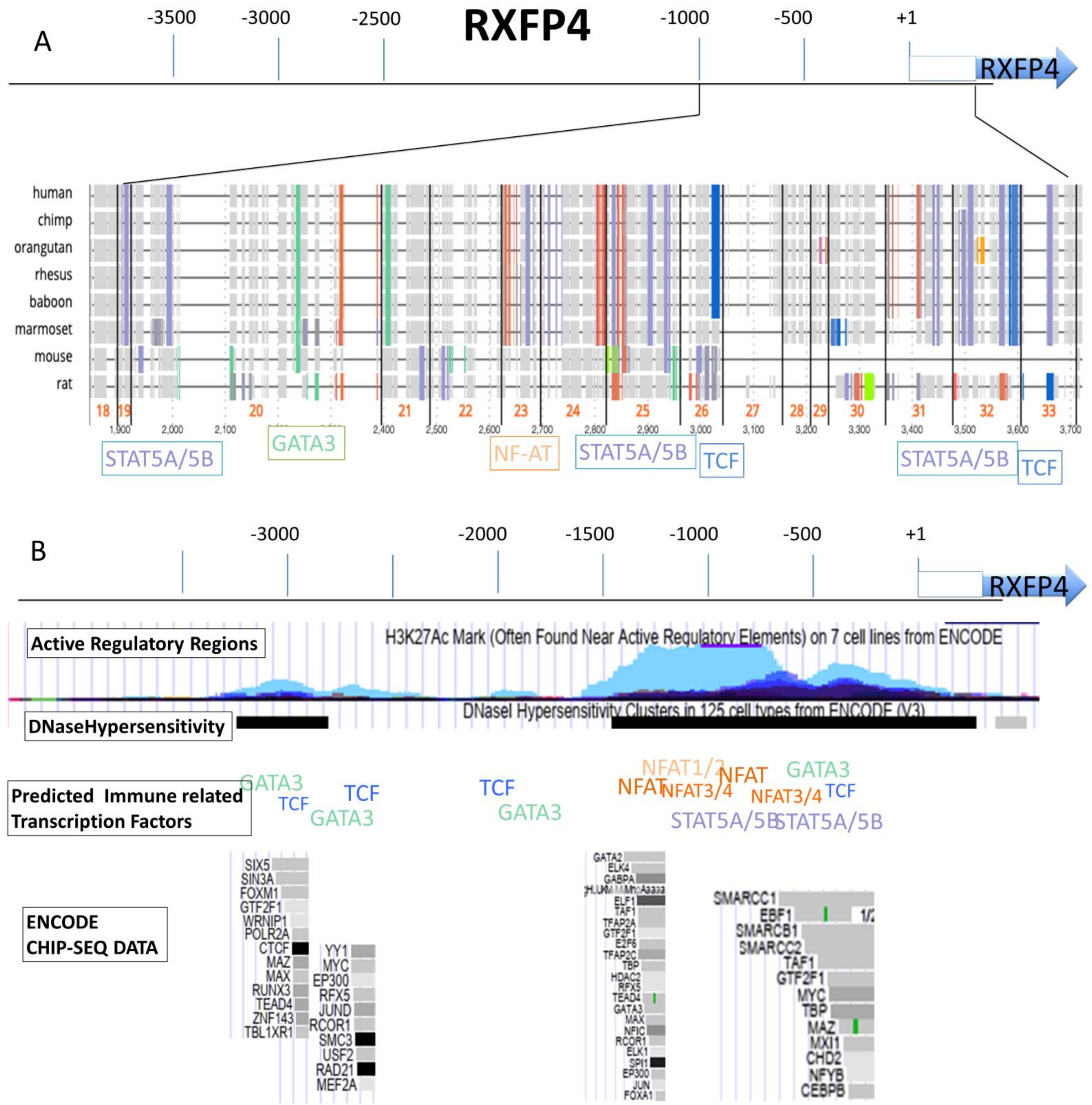

GR

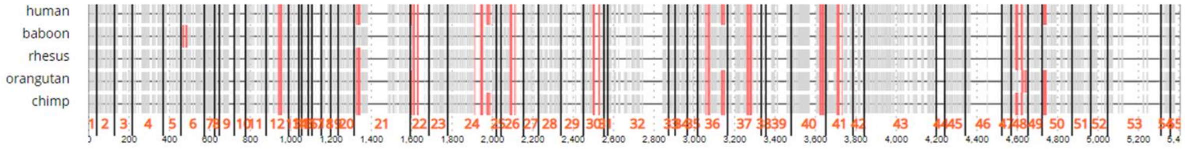

STAT 5a and StAT5b – INSL5

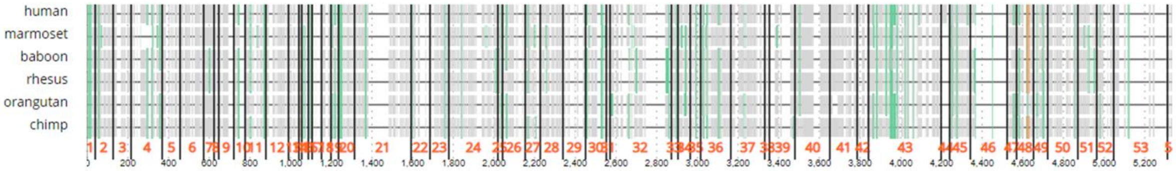

Stat4

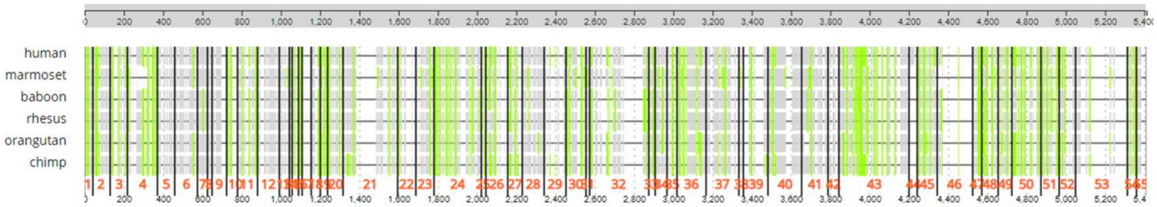

TCF-1, FOXP3

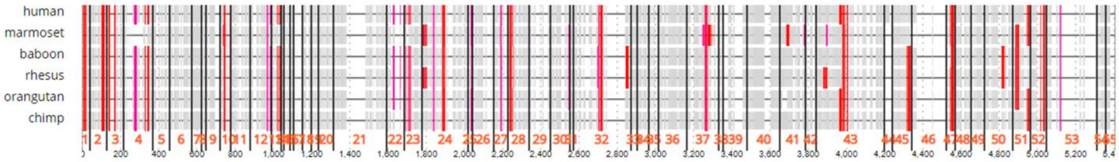

Stat4

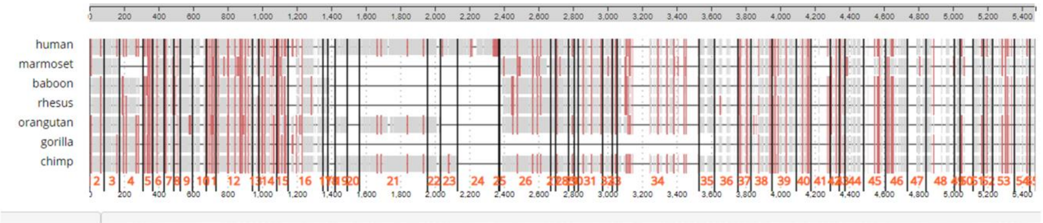

STAT 5a and StAT5b –rxfp4

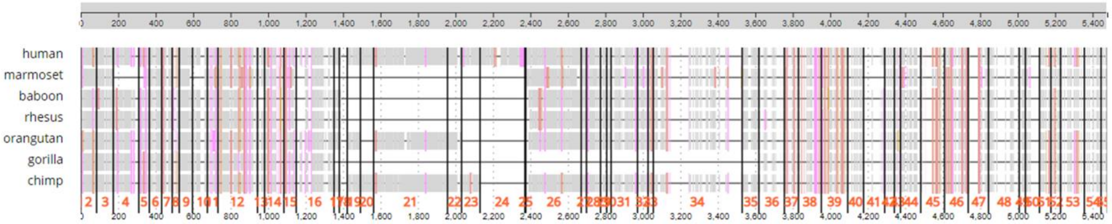

GATA1,2, 3

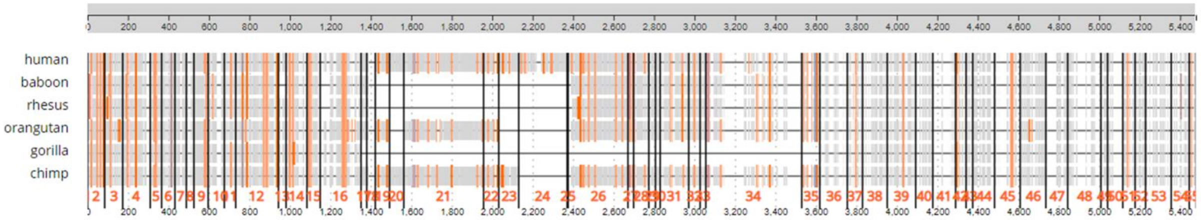

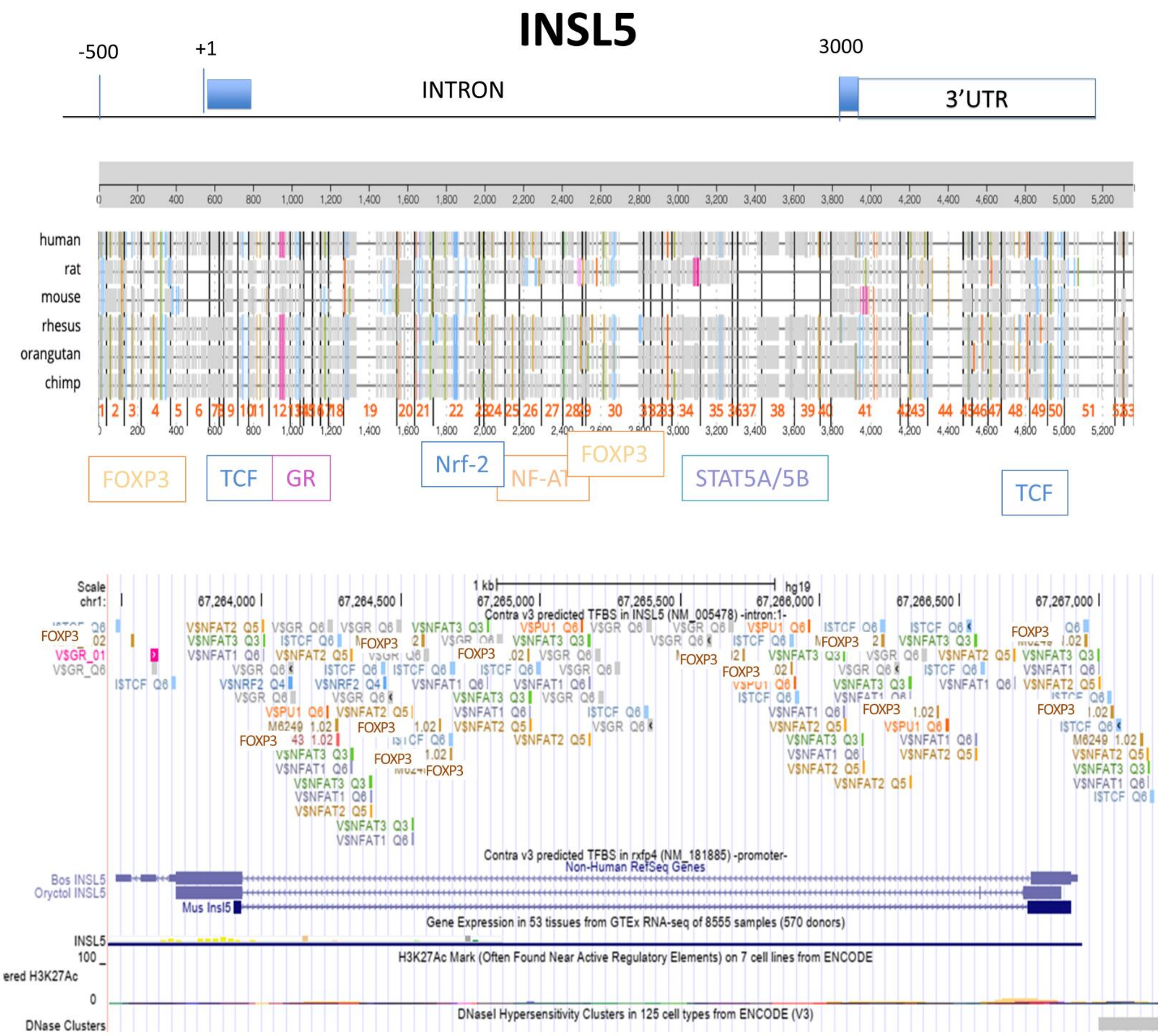
INSL5
-500
+1
3000
INTRON
3'UTR
human
rat
mouse
rhesus
orangutan
chimp
FOXP3
TCF
GR
Nrf-2
NF-AT
FOXP3
STAT5A/5B
TCF
Scale
chr1:
1 kb
hg19
67,264,000
67,264,500
67,265,000
67,265,500
67,266,000
67,266,500
67,267,000
Contra v3 predicted TFBS in INSL5 (NM_005478) -intron:1-
Contra v3 predicted TFBS in rxfp4 (NM_181885) -promoter-
Non-Human RefSeq Genes
Bos INSL5
Oryctol INSL5
Mus Insl5
Gene Expression in 53 tissues from GTEx RNA-seq of 8555 samples (570 donors)
INSL5
100
H3K27Ac Mark (Often Found Near Active Regulatory Elements) on 7 cell lines from ENCODE
ered H3K27Ac
0
DNaseI Hypersensitivity Clusters in 125 cell types from ENCODE (V3)
DNase Clusters
