# Supplementary material for: Immune System Effects of Insulin-Like Peptide 5 in a Mouse Model
Source: Front Endocrinol (Lausanne). 2021 Jan 14;11:610672. doi: 10.3389/fendo.2020.610672 (PMC7841425; doi:10.3389/fendo.2020.610672)
Supplement: Supplementary file 1 [file DataSheet_1.zip › 610672_Supplementary/Supplementary_File_4.PDF]

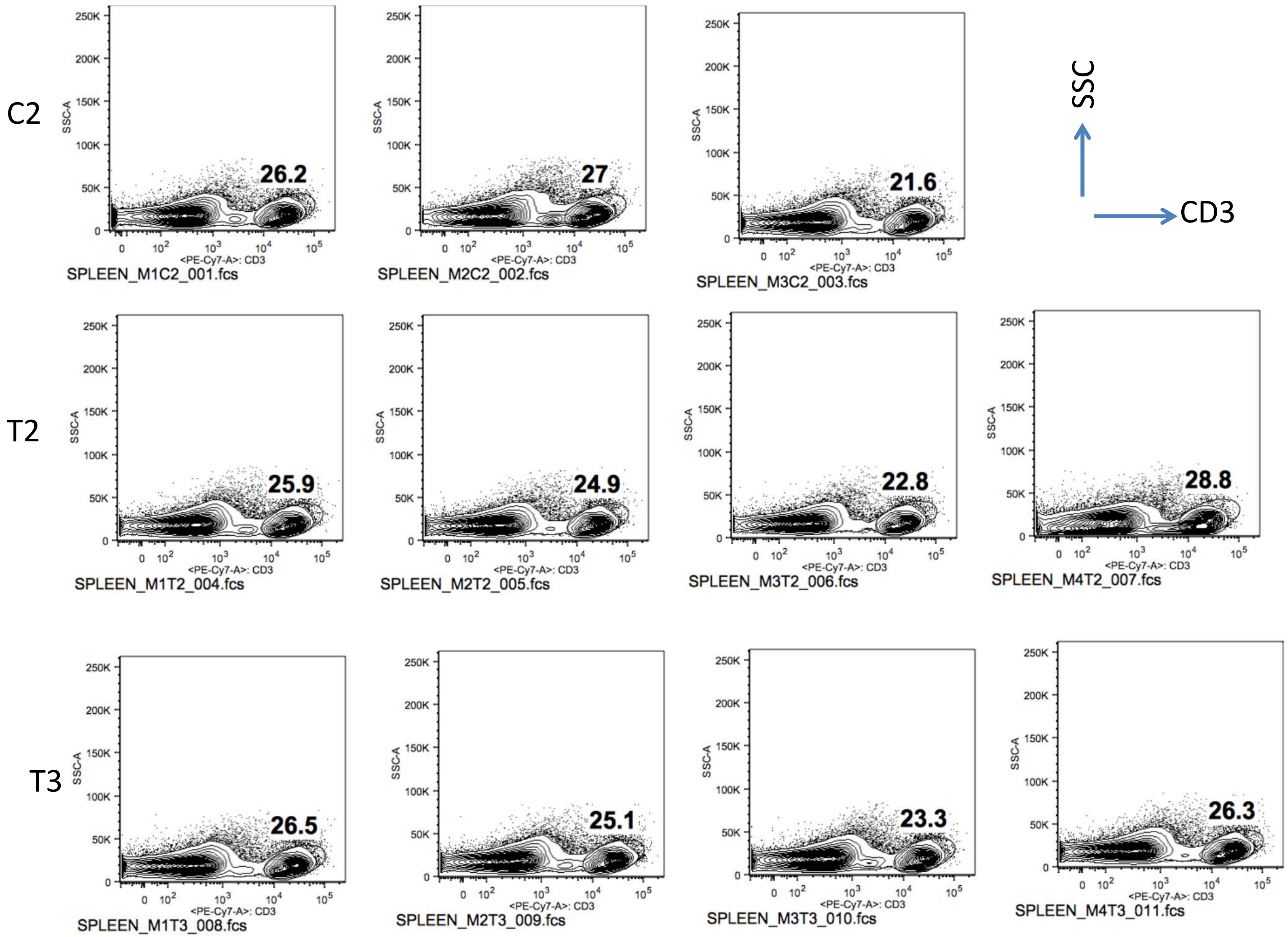

C2
26.2
SPLEEN_M1C2_001.fcs
27
SPLEEN_M2C2_002.fcs
21.6
SPLEEN_M3C2_003.fcs
SSC
CD3
T2
25.9
SPLEEN_M1T2_004.fcs
24.9
SPLEEN_M2T2_005.fcs
22.8
SPLEEN_M3T2_006.fcs
28.8
SPLEEN_M4T2_007.fcs
T3
26.5
SPLEEN_M1T3_008.fcs
25.1
SPLEEN_M2T3_009.fcs
23.3
SPLEEN_M3T3_010.fcs
26.3
SPLEEN_M4T3_011.fcs
SSC-A
<PE-Cy7-A>: CD3

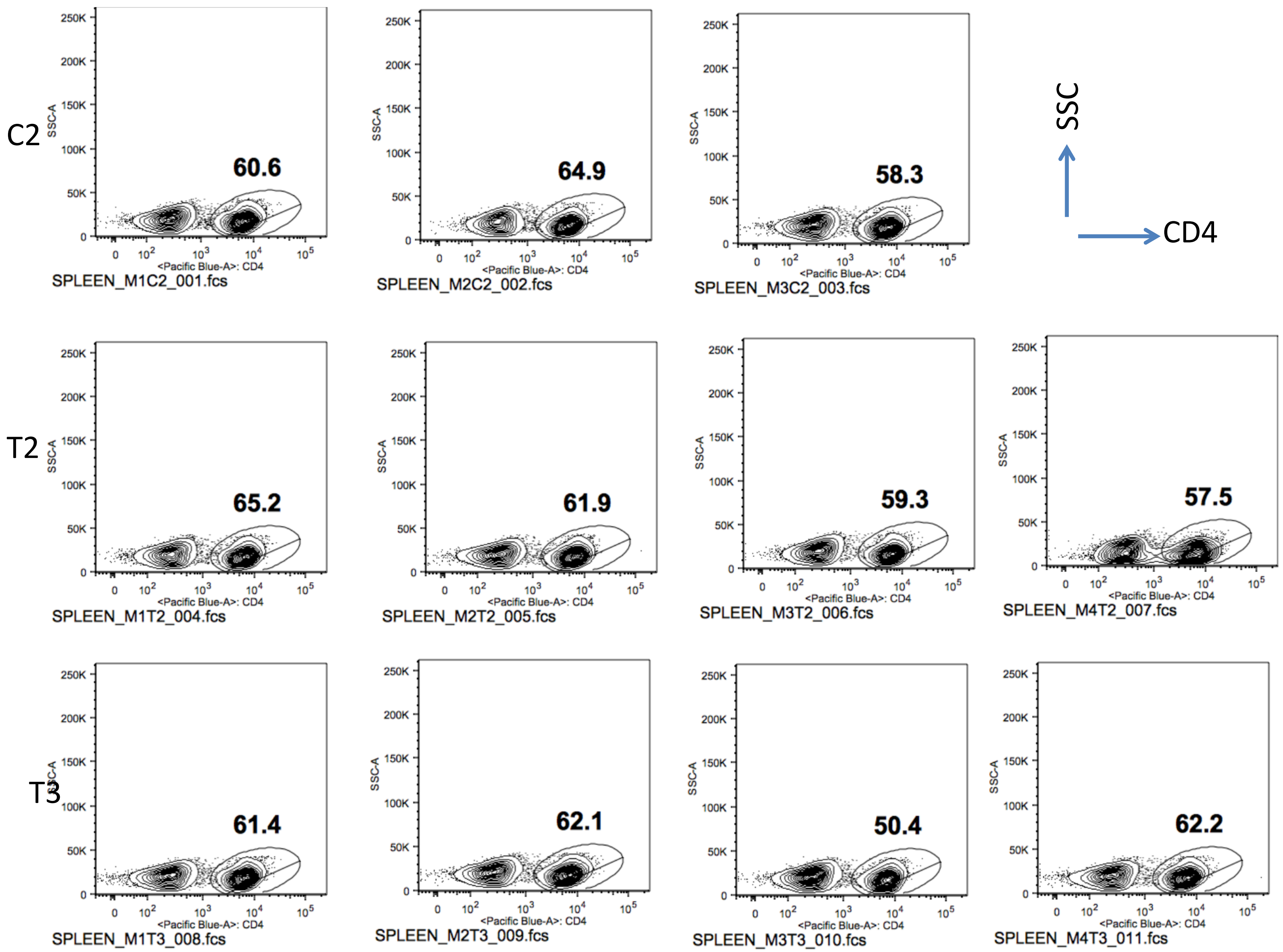
C2
T2
T3
SSC
CD4
SSC-A
<Pacific Blue-A>: CD4
60.6
SPLEEN_M1C2_001.fcs
64.9
SPLEEN_M2C2_002.fcs
58.3
SPLEEN_M3C2_003.fcs
65.2
SPLEEN_M1T2_004.fcs
61.9
SPLEEN_M2T2_005.fcs
59.3
SPLEEN_M3T2_006.fcs
57.5
SPLEEN_M4T2_007.fcs
61.4
SPLEEN_M1T3_008.fcs
62.1
SPLEEN_M2T3_009.fcs
50.4
SPLEEN_M3T3_010.fcs
62.2
SPLEEN_M4T3_011.fcs

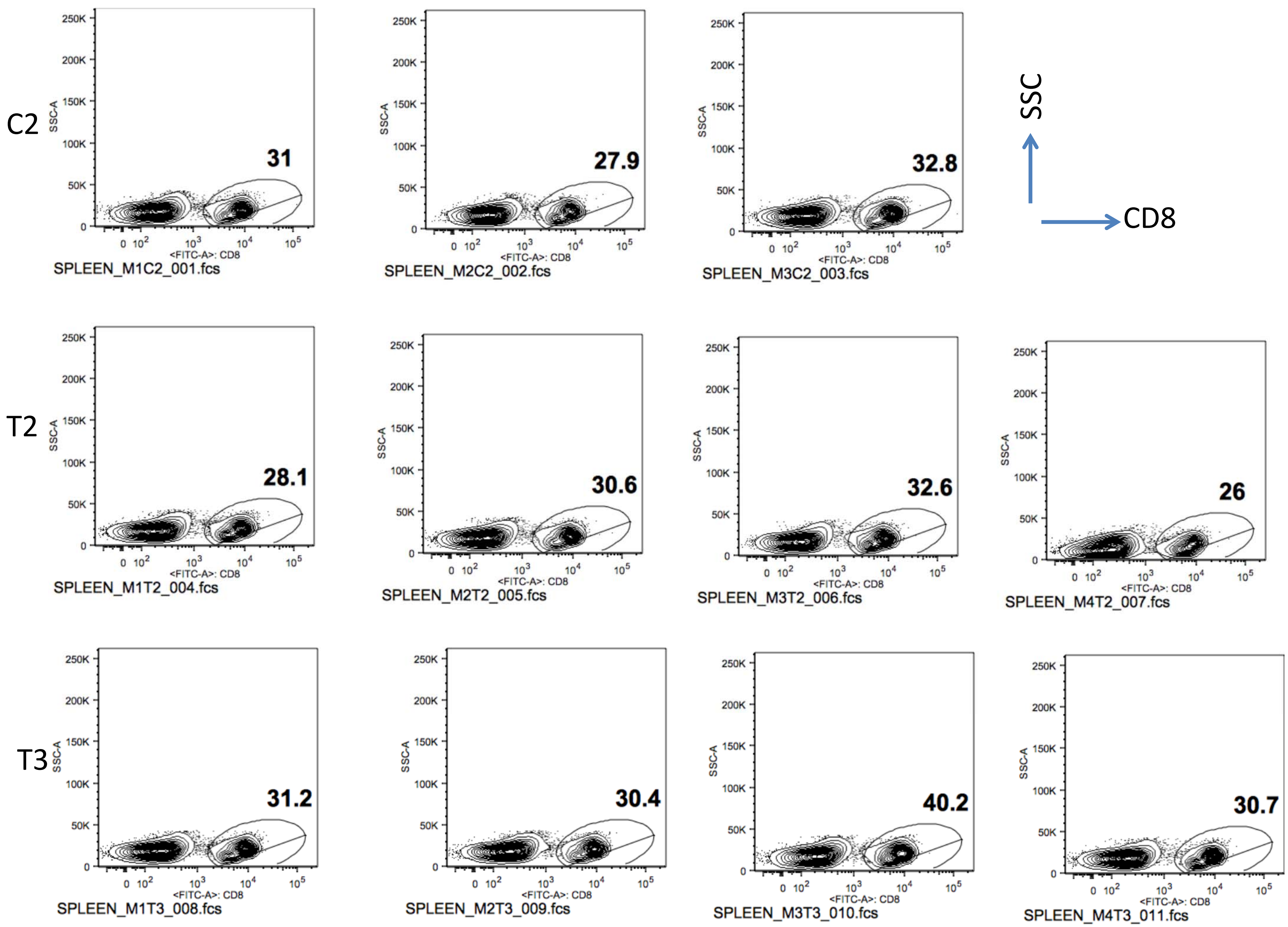
C2
T2
T3
SSC
CD8
31
27.9
32.8
28.1
30.6
32.6
26
31.2
30.4
40.2
30.7
SSC-A
<FITC-A>: CD8
SPLEEN_M1C2_001.fcs
SPLEEN_M2C2_002.fcs
SPLEEN_M3C2_003.fcs
SPLEEN_M1T2_004.fcs
SPLEEN_M2T2_005.fcs
SPLEEN_M3T2_006.fcs
SPLEEN_M4T2_007.fcs
SPLEEN_M1T3_008.fcs
SPLEEN_M2T3_009.fcs
SPLEEN_M3T3_010.fcs
SPLEEN_M4T3_011.fcs

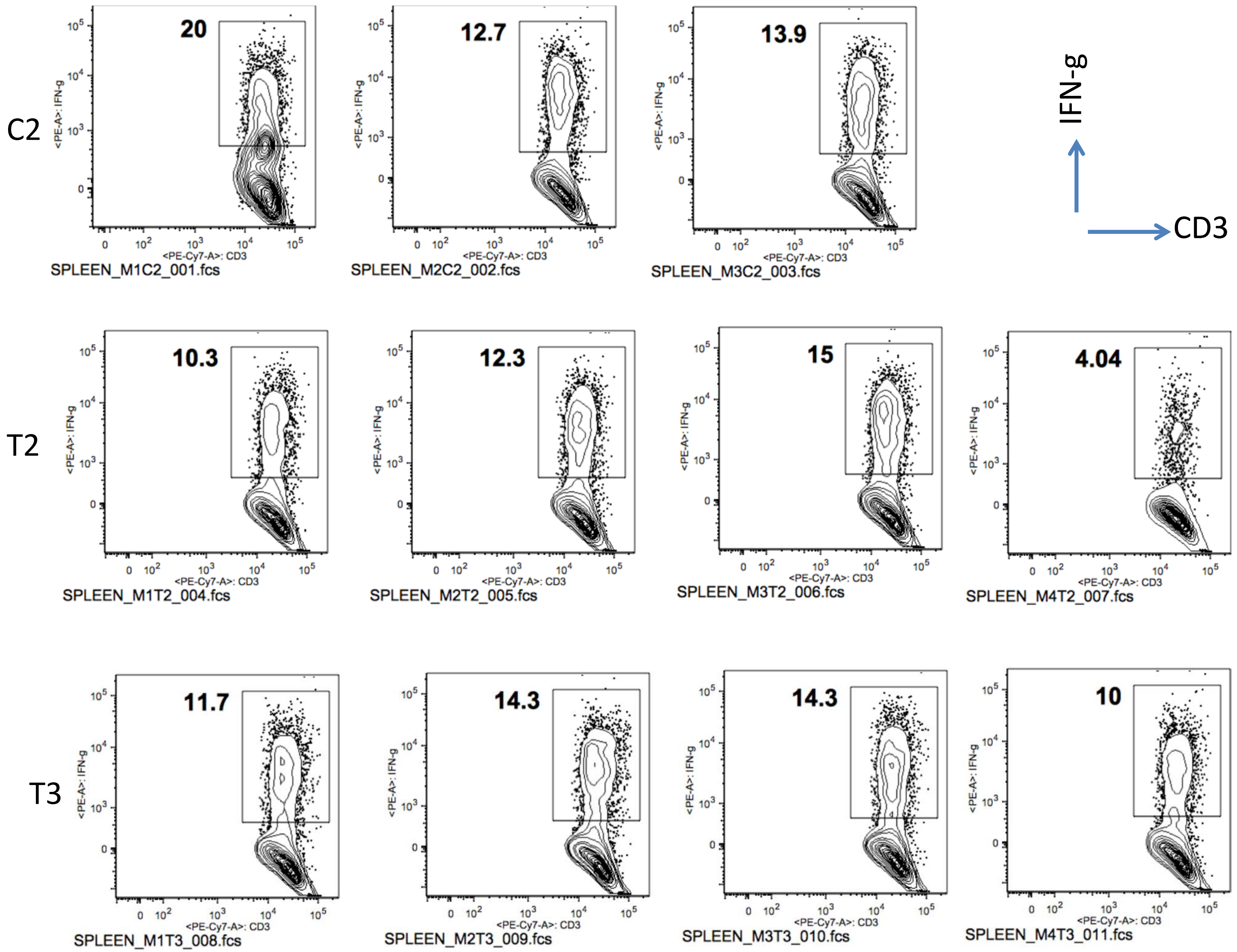
C2
T2
T3
IFN-g
CD3
20
12.7
13.9
10.3
12.3
15
4.04
11.7
14.3
14.3
10
<PE-A>: IFN-g
<PE-Cy7-A>: CD3
SPLEEN_M1C2_001.fcs
SPLEEN_M2C2_002.fcs
SPLEEN_M3C2_003.fcs
SPLEEN_M1T2_004.fcs
SPLEEN_M2T2_005.fcs
SPLEEN_M3T2_006.fcs
SPLEEN_M4T2_007.fcs
SPLEEN_M1T3_008.fcs
SPLEEN_M2T3_009.fcs
SPLEEN_M3T3_010.fcs
SPLEEN_M4T3_011.fcs

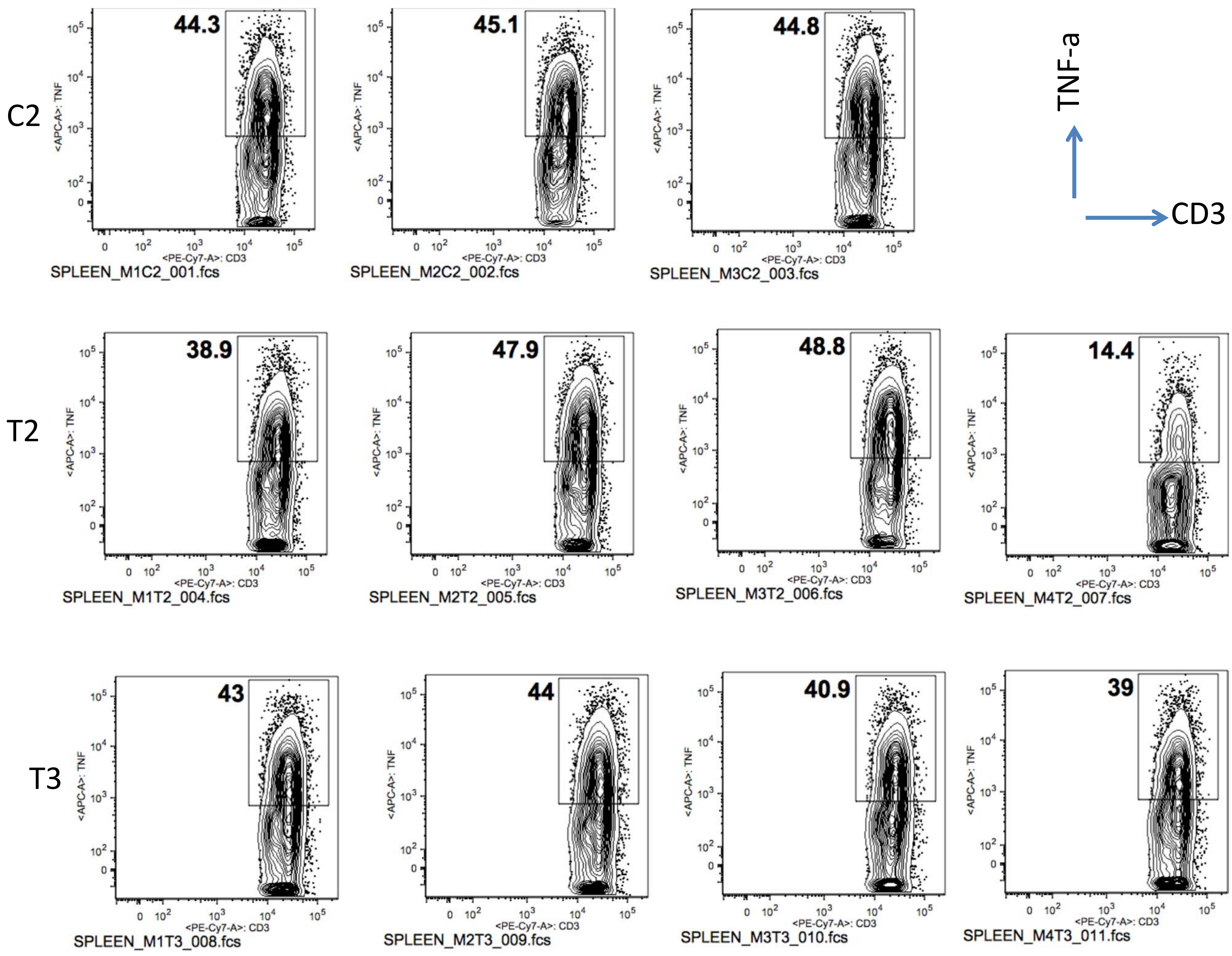

C2
44.3
SPLEEN_M1C2_001.fcs
45.1
SPLEEN_M2C2_002.fcs
44.8
SPLEEN_M3C2_003.fcs
TNF-a
CD3
T2
38.9
SPLEEN_M1T2_004.fcs
47.9
SPLEEN_M2T2_005.fcs
48.8
SPLEEN_M3T2_006.fcs
14.4
SPLEEN_M4T2_007.fcs
T3
43
SPLEEN_M1T3_008.fcs
44
SPLEEN_M2T3_009.fcs
40.9
SPLEEN_M3T3_010.fcs
39
SPLEEN_M4T3_011.fcs
<APC-A>: TNF
<PE-Cy7-A>: CD3

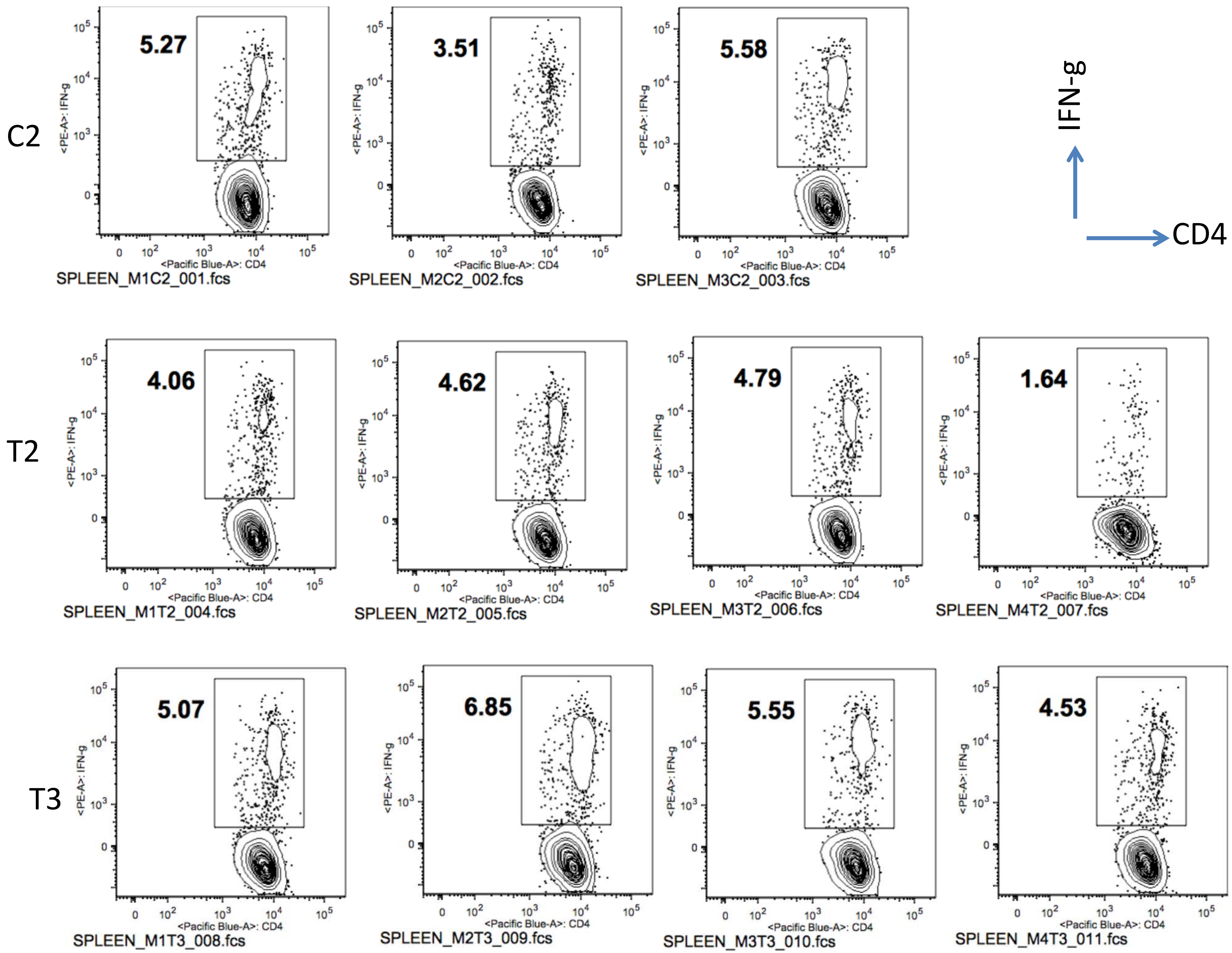

C2
T2
T3
IFN-g
CD4
5.27
3.51
5.58
4.06
4.62
4.79
1.64
5.07
6.85
5.55
4.53
<PE-A>: IFN-g
<Pacific Blue-A>: CD4
SPLEEN_M1C2_001.fcs
SPLEEN_M2C2_002.fcs
SPLEEN_M3C2_003.fcs
SPLEEN_M1T2_004.fcs
SPLEEN_M2T2_005.fcs
SPLEEN_M3T2_006.fcs
SPLEEN_M4T2_007.fcs
SPLEEN_M1T3_008.fcs
SPLEEN_M2T3_009.fcs
SPLEEN_M3T3_010.fcs
SPLEEN_M4T3_011.fcs

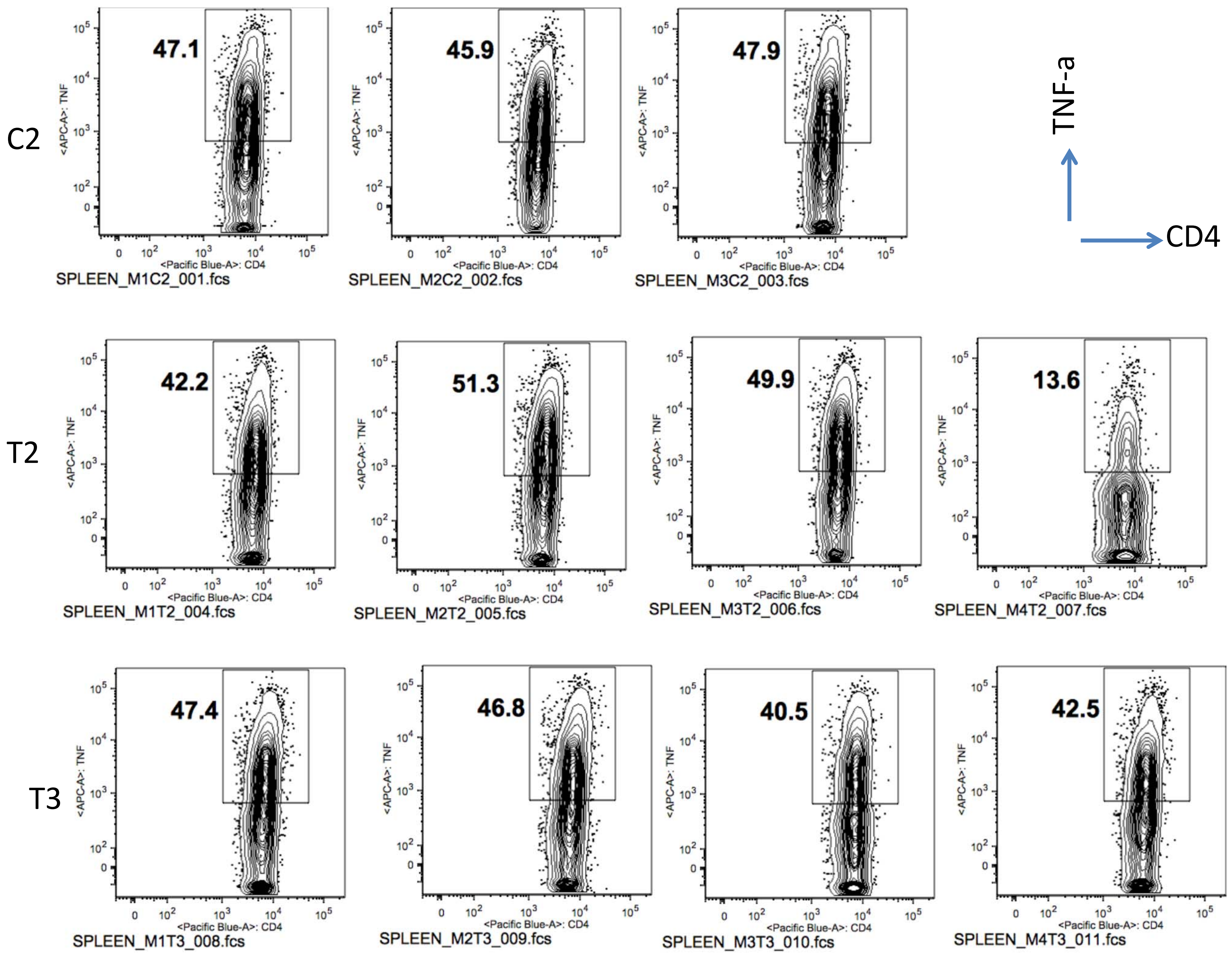
C2
T2
T3
TNF-a
CD4
47.1
45.9
47.9
42.2
51.3
49.9
13.6
47.4
46.8
40.5
42.5
<APC-A>: TNF
<Pacific Blue-A>: CD4
SPLEEN_M1C2_001.fcs
SPLEEN_M2C2_002.fcs
SPLEEN_M3C2_003.fcs
SPLEEN_M1T2_004.fcs
SPLEEN_M2T2_005.fcs
SPLEEN_M3T2_006.fcs
SPLEEN_M4T2_007.fcs
SPLEEN_M1T3_008.fcs
SPLEEN_M2T3_009.fcs
SPLEEN_M3T3_010.fcs
SPLEEN_M4T3_011.fcs

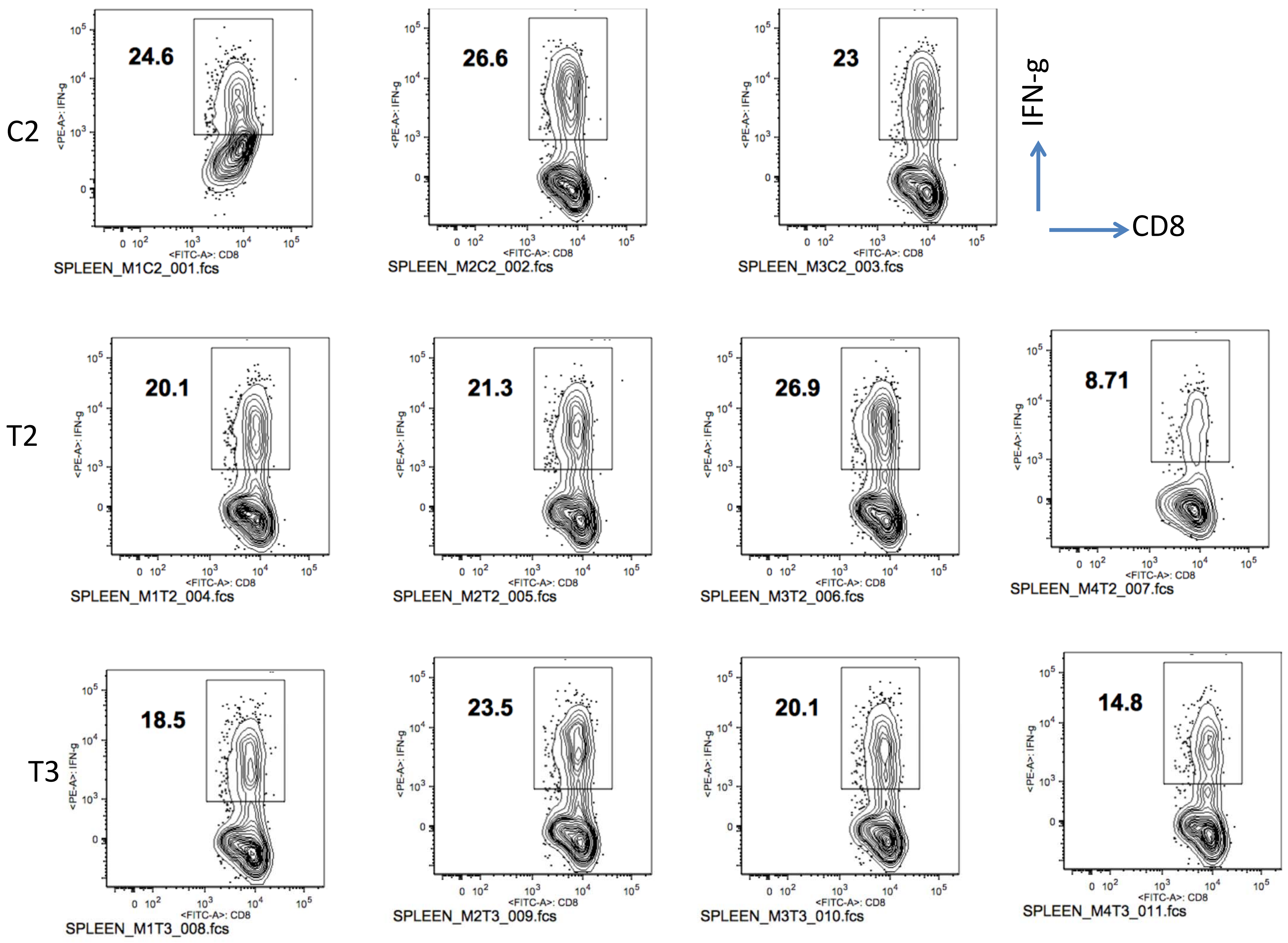
C2
T2
T3
IFN-g
CD8
24.6
26.6
23
20.1
21.3
26.9
8.71
18.5
23.5
20.1
14.8
<PE-A>: IFN-g
<FITC-A>: CD8
SPLEEN_M1C2_001.fcs
SPLEEN_M2C2_002.fcs
SPLEEN_M3C2_003.fcs
SPLEEN_M1T2_004.fcs
SPLEEN_M2T2_005.fcs
SPLEEN_M3T2_006.fcs
SPLEEN_M4T2_007.fcs
SPLEEN_M1T3_008.fcs
SPLEEN_M2T3_009.fcs
SPLEEN_M3T3_010.fcs
SPLEEN_M4T3_011.fcs

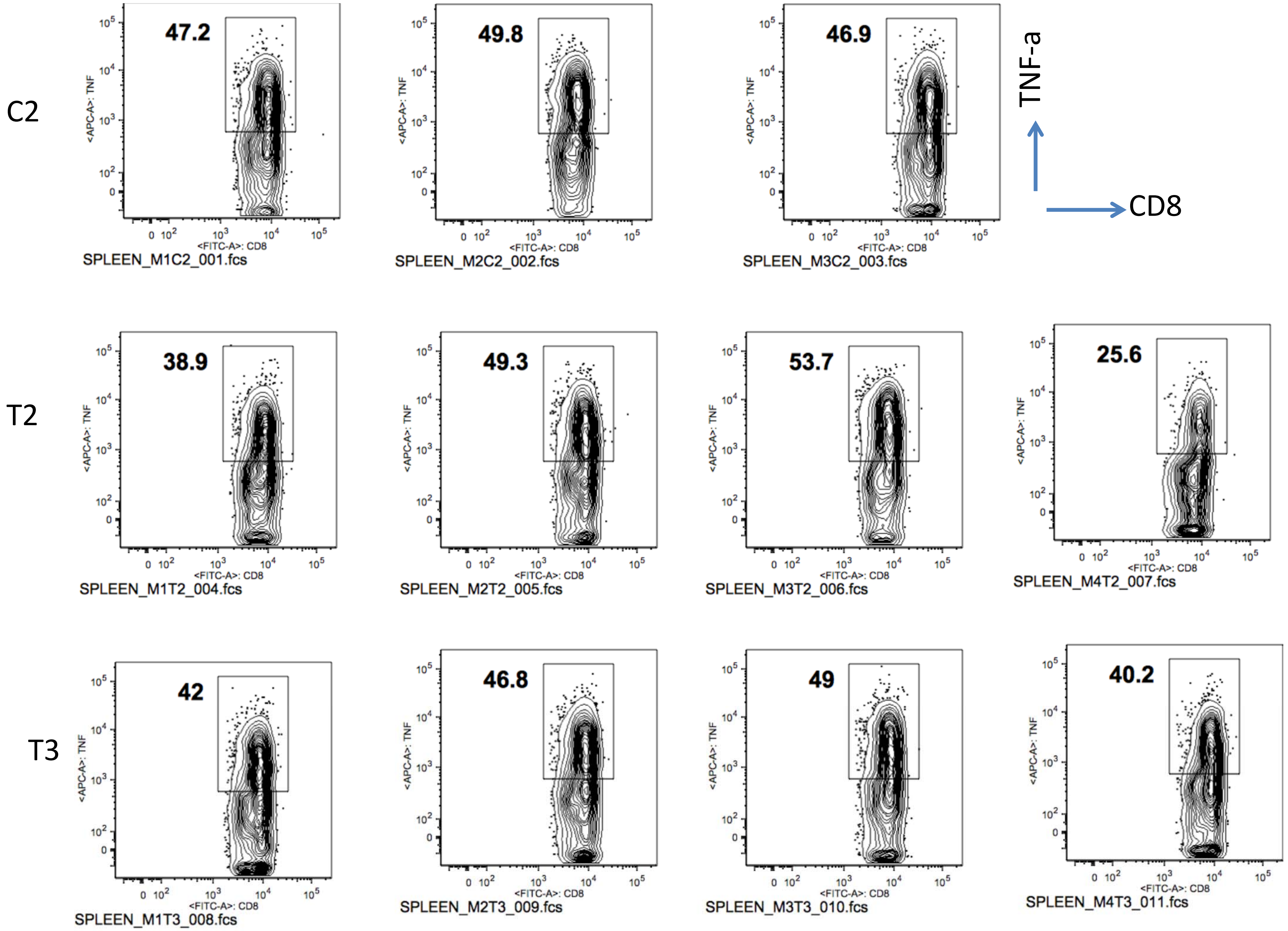
C2
T2
T3
TNF-a
CD8
47.2
49.8
46.9
38.9
49.3
53.7
25.6
42
46.8
49
40.2
<APC-A>: TNF
<FITC-A>: CD8
SPLEEN_M1C2_001.fcs
SPLEEN_M2C2_002.fcs
SPLEEN_M3C2_003.fcs
SPLEEN_M1T2_004.fcs
SPLEEN_M2T2_005.fcs
SPLEEN_M3T2_006.fcs
SPLEEN_M4T2_007.fcs
SPLEEN_M1T3_008.fcs
SPLEEN_M2T3_009.fcs
SPLEEN_M3T3_010.fcs
SPLEEN_M4T3_011.fcs

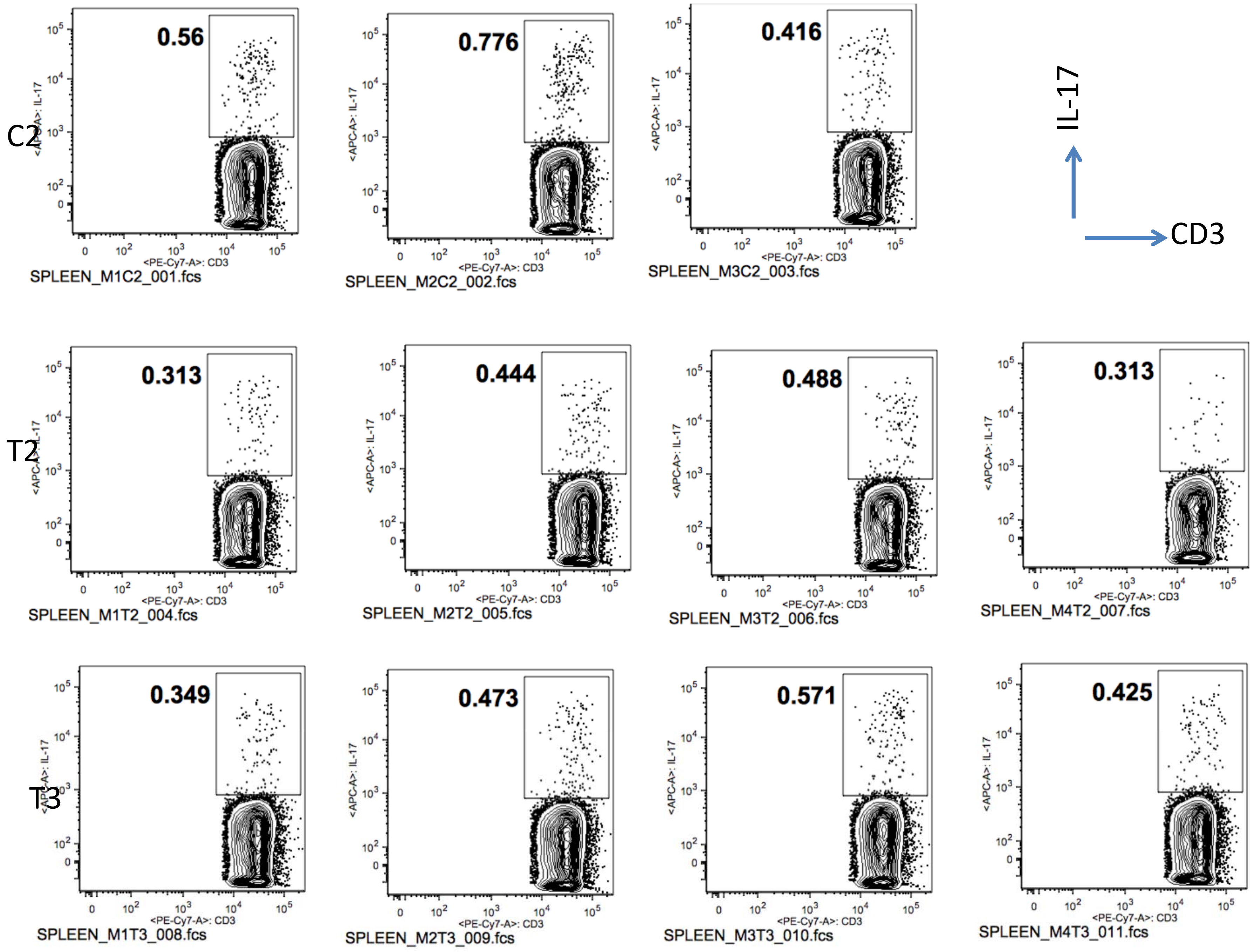

IL-17
CD3
C2
T2
T3
0.56
SPLEEN_M1C2_001.fcs
0.776
SPLEEN_M2C2_002.fcs
0.416
SPLEEN_M3C2_003.fcs
0.313
SPLEEN_M1T2_004.fcs
0.444
SPLEEN_M2T2_005.fcs
0.488
SPLEEN_M3T2_006.fcs
0.313
SPLEEN_M4T2_007.fcs
0.349
SPLEEN_M1T3_008.fcs
0.473
SPLEEN_M2T3_009.fcs
0.571
SPLEEN_M3T3_010.fcs
0.425
SPLEEN_M4T3_011.fcs
<APC-A>: IL-17
<PE-Cy7-A>: CD3

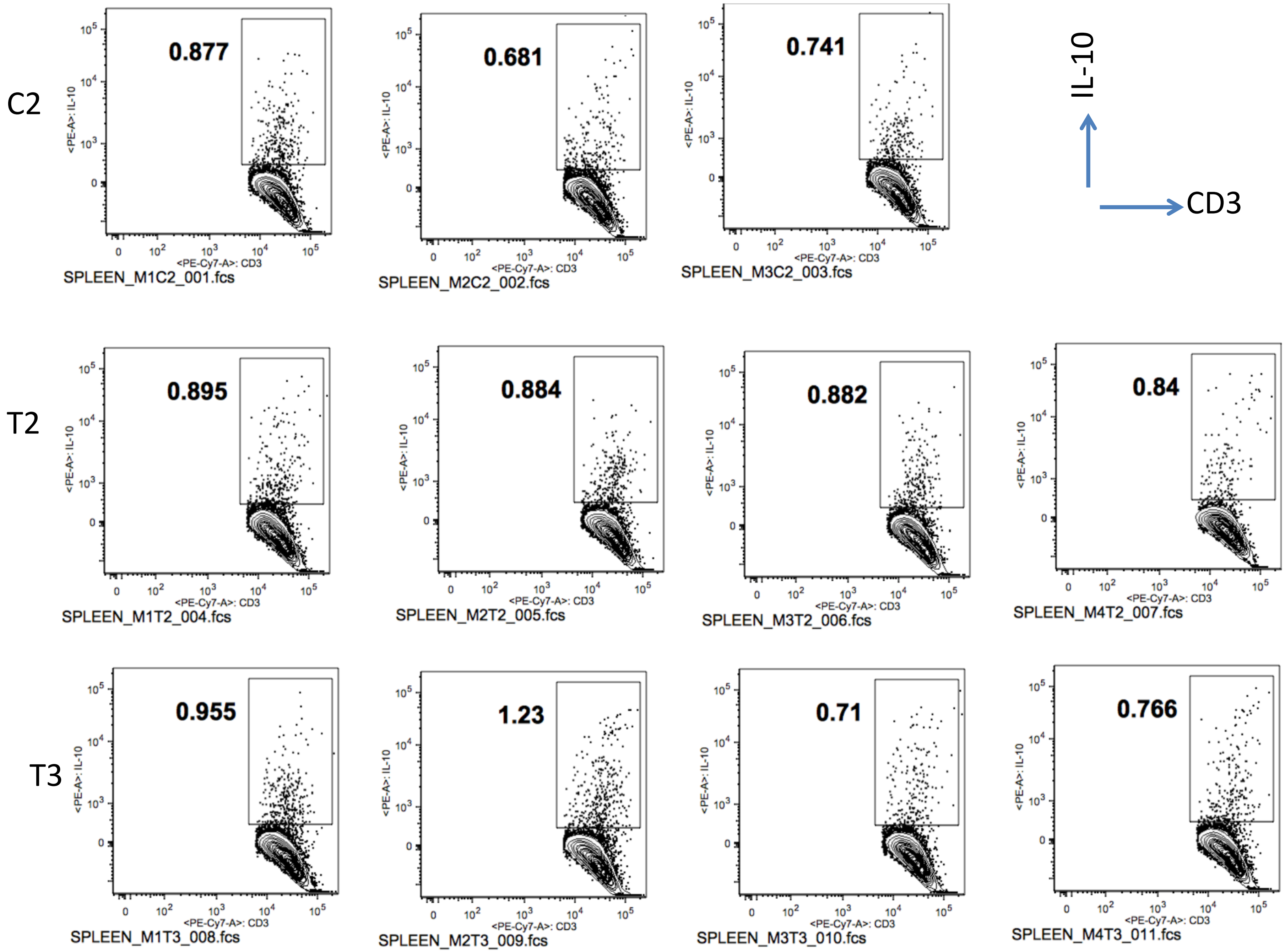
C2
0.877
SPLEEN_M1C2_001.fcs
0.681
SPLEEN_M2C2_002.fcs
0.741
SPLEEN_M3C2_003.fcs
IL-10
CD3
T2
0.895
SPLEEN_M1T2_004.fcs
0.884
SPLEEN_M2T2_005.fcs
0.882
SPLEEN_M3T2_006.fcs
0.84
SPLEEN_M4T2_007.fcs
T3
0.955
SPLEEN_M1T3_008.fcs
1.23
SPLEEN_M2T3_009.fcs
0.71
SPLEEN_M3T3_010.fcs
0.766
SPLEEN_M4T3_011.fcs
<PE-A>: IL-10
<PE-Cy7-A>: CD3

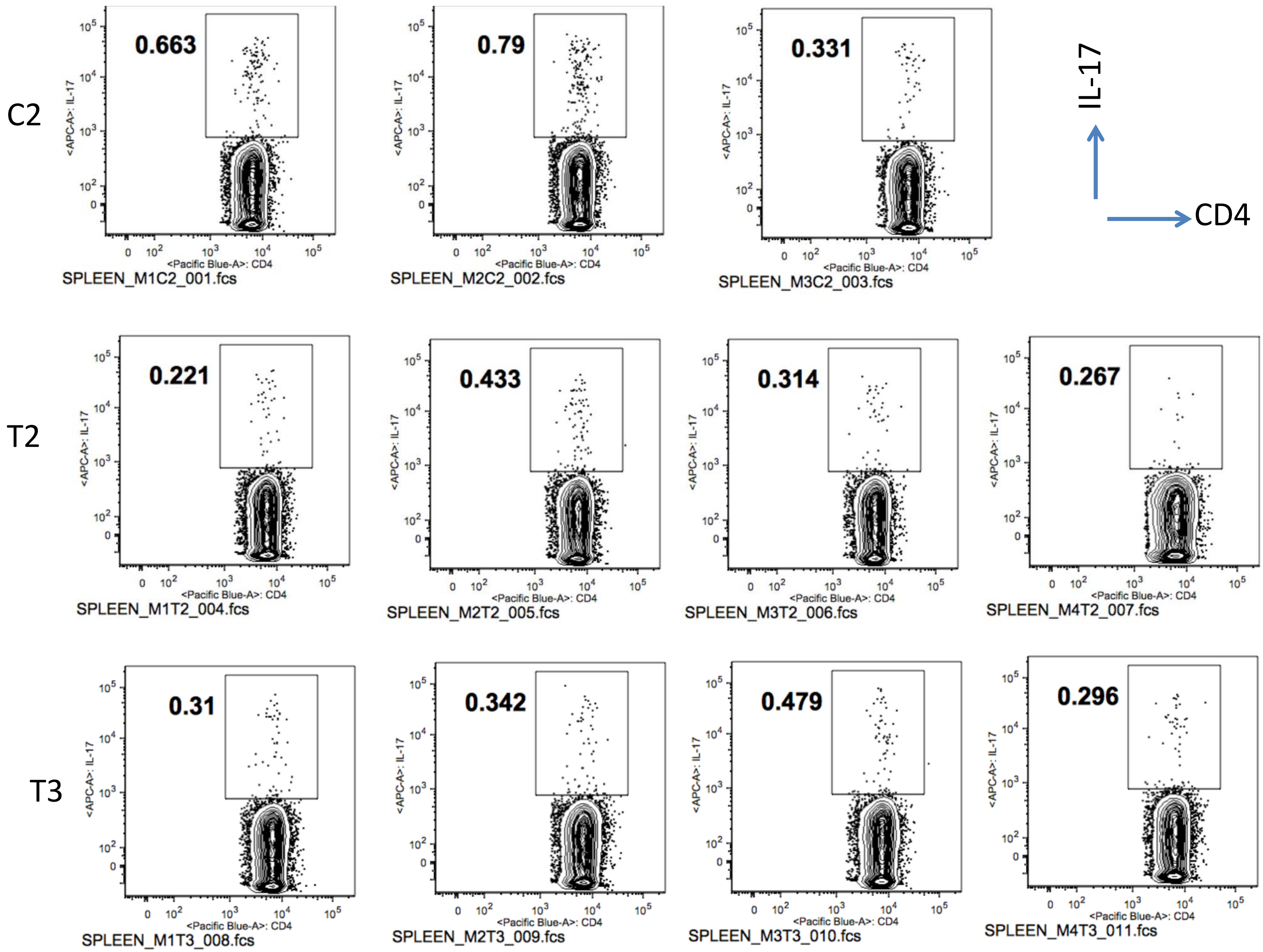

C2
T2
T3
IL-17
CD4
0.663
0.79
0.331
0.221
0.433
0.314
0.267
0.31
0.342
0.479
0.296
<APC-A>: IL-17
<Pacific Blue-A>: CD4
SPLEEN_M1C2_001.fcs
SPLEEN_M2C2_002.fcs
SPLEEN_M3C2_003.fcs
SPLEEN_M1T2_004.fcs
SPLEEN_M2T2_005.fcs
SPLEEN_M3T2_006.fcs
SPLEEN_M4T2_007.fcs
SPLEEN_M1T3_008.fcs
SPLEEN_M2T3_009.fcs
SPLEEN_M3T3_010.fcs
SPLEEN_M4T3_011.fcs

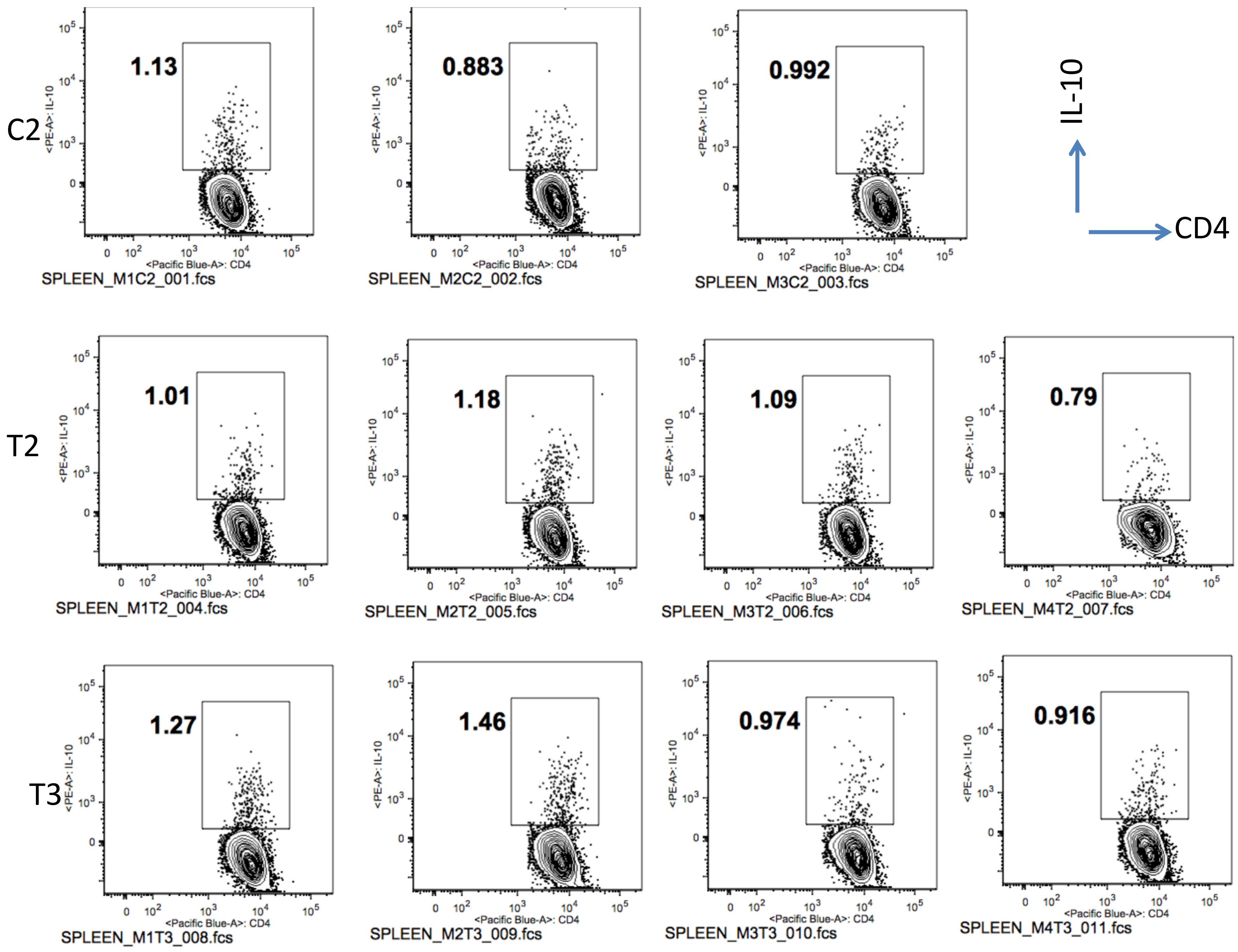

C2
T2
T3
IL-10
CD4
1.13
0.883
0.992
1.01
1.18
1.09
0.79
1.27
1.46
0.974
0.916
<PE-A>: IL-10
<Pacific Blue-A>: CD4
SPLEEN_M1C2_001.fcs
SPLEEN_M2C2_002.fcs
SPLEEN_M3C2_003.fcs
SPLEEN_M1T2_004.fcs
SPLEEN_M2T2_005.fcs
SPLEEN_M3T2_006.fcs
SPLEEN_M4T2_007.fcs
SPLEEN_M1T3_008.fcs
SPLEEN_M2T3_009.fcs
SPLEEN_M3T3_010.fcs
SPLEEN_M4T3_011.fcs

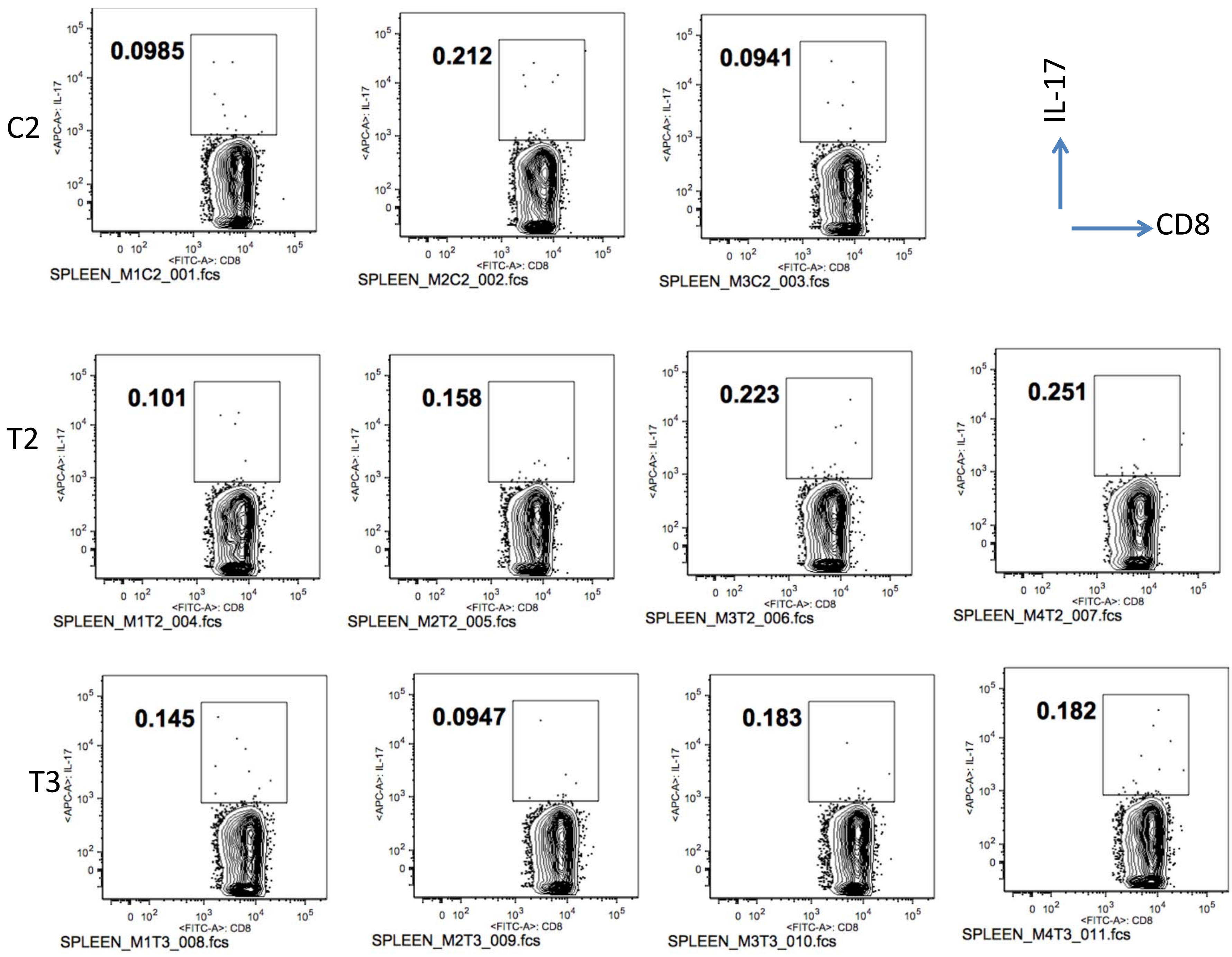

C2
T2
T3
IL-17
CD8
0.0985
0.212
0.0941
0.101
0.158
0.223
0.251
0.145
0.0947
0.183
0.182
<APC-A>: IL-17
<FITC-A>: CD8
SPLEEN_M1C2_001.fcs
SPLEEN_M2C2_002.fcs
SPLEEN_M3C2_003.fcs
SPLEEN_M1T2_004.fcs
SPLEEN_M2T2_005.fcs
SPLEEN_M3T2_006.fcs
SPLEEN_M4T2_007.fcs
SPLEEN_M1T3_008.fcs
SPLEEN_M2T3_009.fcs
SPLEEN_M3T3_010.fcs
SPLEEN_M4T3_011.fcs

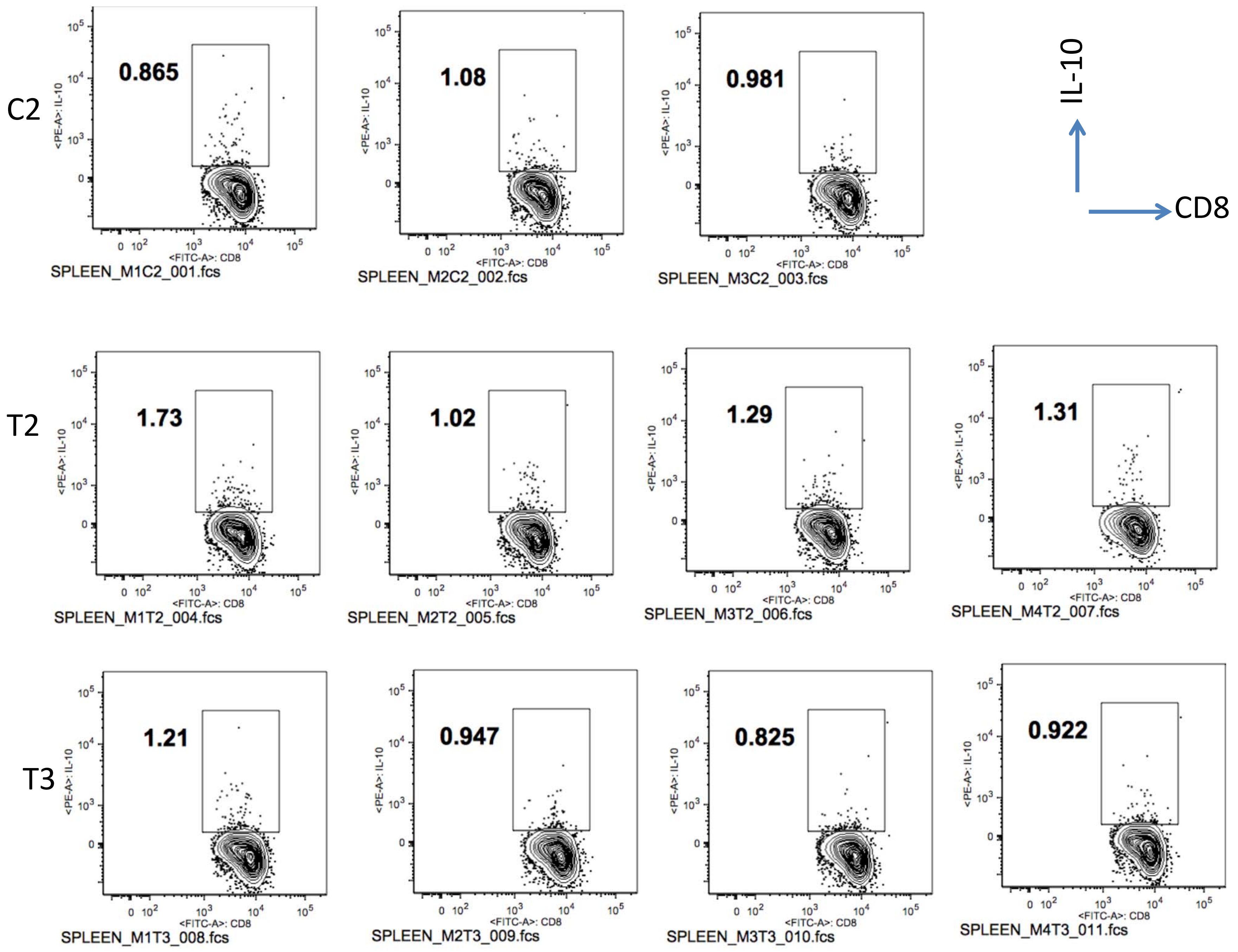

C2
T2
T3
IL-10
CD8
0.865
1.08
0.981
1.73
1.02
1.29
1.31
1.21
0.947
0.825
0.922
<PE-A>: IL-10
<FITC-A>: CD8
SPLEEN_M1C2_001.fcs
SPLEEN_M2C2_002.fcs
SPLEEN_M3C2_003.fcs
SPLEEN_M1T2_004.fcs
SPLEEN_M2T2_005.fcs
SPLEEN_M3T2_006.fcs
SPLEEN_M4T2_007.fcs
SPLEEN_M1T3_008.fcs
SPLEEN_M2T3_009.fcs
SPLEEN_M3T3_010.fcs
SPLEEN_M4T3_011.fcs
