# Supplementary material for: Immune System Effects of Insulin-Like Peptide 5 in a Mouse Model
Source: Front Endocrinol (Lausanne). 2021 Jan 14;11:610672. doi: 10.3389/fendo.2020.610672 (PMC7841425; doi:10.3389/fendo.2020.610672)
Supplement: Supplementary file 1 [file DataSheet_1.zip › 610672_Supplementary/Supplementary_File_5.PDF]

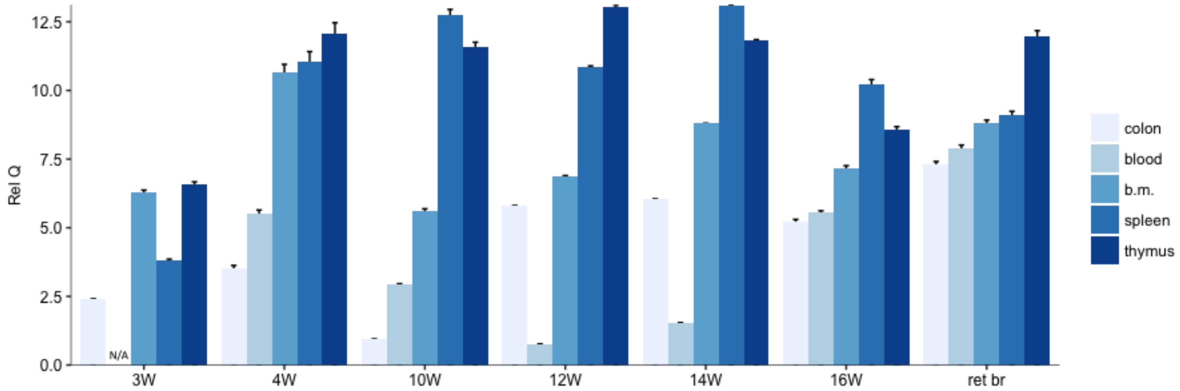

Figure S1. Spatial change in *rxfp4* gene expression in C57BL6 male colon, blood, bone marrow (b.m.), spleen and thymus, where log2 (Rel Q) expression values shown on y-axis represent calibration of *Rxfp4* expression value to blood of a 3-week-old mouse, indicated by N/A on the graph. Mouse ages of 3, 4, 10, 12, 14, 16 weeks and retired breeders are shown on x-axis.

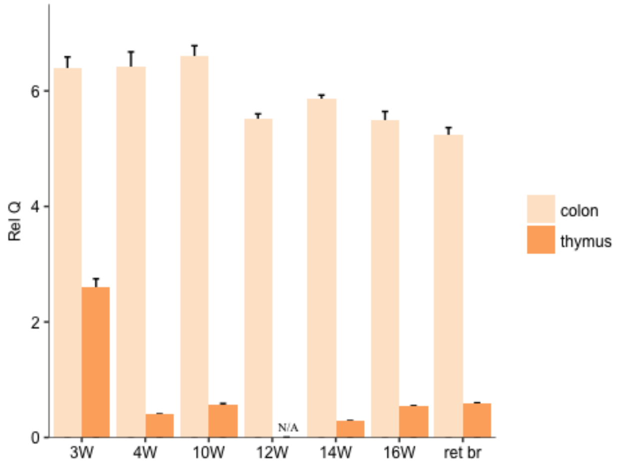

Figure S2. Spatial change in *insl5* gene expression in C57BL6 male colon and thymus, where log2 (Rel Q) expression values shown on the y-axis represent normalization of expression in both tissues to the expression value in thymus at 12 weeks, indicated by N/A on the graph. Mouse ages of 3, 4, 10, 12, 14, 16 weeks and retired breeders are shown on x-axis. No signal was detected for *insl5* transcript in blood, bone marrow or spleen.
